# What gets measured in palliative care? A review and synthesis of routine data collection in 16 countries

**DOI:** 10.1016/j.hpopen.2025.100141

**Published:** 2025-04-19

**Authors:** Eimir Hurley, Peter May, Soraya Matthews, Charles Normand, Bridget M. Johnston

**Affiliations:** a Centre for Health Policy and Management, Trinity College Dublin, University of Dublin, 2-4 Foster Place, Dublin D2, Ireland; b The Irish Longitudinal Study on Ageing, Trinity College Dublin, University of Dublin, College Green, Dublin D2, Ireland; c Cicely Saunders Institute of Palliative Care, Policy & Rehabilitation, King’s College London, Bessemer Road, London SE5 9PJ England, UK

**Keywords:** Big data, Palliative care, Health care systems, Comparative review

## Abstract

**Background:**

There is an increasing focus on strengthening palliative care data infrastructure to evaluate and improve the quality of care. We conducted an extensive review of policy documents to identify international best practice in the use of routine data in palliative care.

**Methods:**

We identified 16 countries with well-established palliative care services before undertaking the review. We searched systematically for relevant documentation on each country in the academic, grey and governmental literature. For each country we then compiled a narrative synthesis utilising a standardised extraction template. Local experts verified country-level synopses. We combined the 16 country documents using thematic synthesis.

**Results:**

There was significant heterogeneity in the data infrastructure of the countries examined. The majority of the databases and data sources focused on specialist palliative care services with a notable lack of data on palliative care delivered in primary and community care. Several countries have established bespoke palliative care databases; others harness existing data sources, and capitalise on the existence of unique patient identifiers. The gaps and limitations identified were commonly shared across all types of palliative and end of life care data infrastructure. Similarly, many of the factors deemed highly influential in implementing and sustaining existing databases are relevant across all data infrastructure.

**Conclusions:**

This first-of-its-kind analysis details the characteristics of databases/data sources and highlights the significant heterogeneity which exists. The strengths and limitations of existing databases/data sources and the factors that influence how well these systems are sustained are examined, providing key learnings for those eager to improve the data infrastructure in their own jurisdictions.

## 1 Introduction

### 1.1 Background and rationale

Strong data infrastructure has long been recognised as essential in health policy and planning for several purposes, including documenting patterns of supply and realised demand; quantifying unmet needs; and evaluating the effectiveness and cost-effectiveness of services [1]. Health care, as with the rest of society, has now entered an era of ‘big data’, in which large volumes of information are collected routinely [2].

‘Big’ data may be particularly valuable in guiding the development of palliative and end of life care provision (hereafter, PEoLC), where the evidence base remains limited due to the ethical and practical challenges conducting primary research studies including ensuring adequate recruitment and retention, and difficulties collecting data on relevant outcomes [[3], [4], [5]]. Palliative care is an approach “that improves the quality of life of patients and their families facing the problems associated with life-threatening illness, through the prevention and relief of suffering by means of early identification and impeccable assessment and treatment of pain and other problems, physical, psychosocial and spiritual” [6]. Studies indicate that palliative care improves outcomes and can reduce health care costs for people with serious illness [5]. In the context of well-documented population health needs in the 21st century, increased palliative care capacity is required worldwide []. Policy and practice must be informed by the best available evidence, including routinely collected data on PEoLC provision and outcomes [6].

To our knowledge, there has been no systematic analysis that has mapped and described existing datasets and data sources (data infrastructure) in the domain of PEoLC across a range of high-income countries. Additionally, international research has not collated information about how these datasets and sources are used to inform policy and practice, or identified the factors that promote or hinder their implementation and sustainability.

### 1.2 Aims

The aim of this research was to synthesise international current practice and experiences with respect to the collection and use of routine PEoLC data in planning and evaluation. In this analysis, ‘PEoLC data’ is defined as routinely collected data resources specific to palliative and/or end of life care provision and outcomes, including administrative data, data collections and national registries. Additionally, the term ‘PEoLC database’ is used to describe an organised collection of data specific to PEoLC, that is stored electronically. We selected 16 countries with well-established palliative care services by international standards and universal health care systems. We carried out searches to identify relevant documentation for each of these countries, and we engaged local experts in each country to check the accuracy and comprehensiveness of our results. We synthesised these results thematically to address four objectives:

1. To describe the data sources which constitute the PEoLC data infrastructure in the selected countries and how these are operationalised
2. To examine how the PEoLC data infrastructure is used in informing palliative care service planning and care delivery;
3. To document the recognised limitations of the existing PEoLC data infrastructure in the countries examined and chart current and future efforts to address these limitations;
4. To identify the factors that influence the implementation and sustainability of existing PEoLC data infrastructure.

## 2 Methods

### 2.1 Study design

This was a thematic synthesis study. We identified relevant documentation for each country by searching in the academic, grey and governmental literature and compiled extracted data into a narrative synthesis for each country. We shared each country-specific synthesis with a local expert who checked this work for accuracy, understanding and completeness. Following expert input, we revised and added material as required, and sought expert approval for each revised country-specific synthesis. Finally, we combined the 16 country documents using thematic synthesis according to identified themes.

#### 2.1.1 Countries studied

We focused on high-income countries with universal health care systems and well-established palliative care services. This choice was guided by the ‘Knowledge User’ partner, which was the palliative care integration and planning programme within the Health Service Executive, the publicly funded healthcare system in the Republic of Ireland (Further details of the grant award are provided in the Funding section). The ‘Knowledge User’ had no influence over the analysis, interpretation or reporting of this study.

To identify countries, we cross-referenced the Global Atlas of Palliative Care at the End of life, with Organisation for Economic Co-operation and Development (OECD) member countries, and included those countries ranked 4a or 4b in the Global Atlas, and additionally ranked in the top 20 of the Economist Quality of Death Index [8,9]. Sixteen countries met these eligibility criteria: Australia, Austria, Belgium, Canada, Denmark, Finland, France, Germany, Ireland, Japan, The Netherlands, New Zealand, Norway, Sweden, Switzerland and the United Kingdom (comprising England, Northern Ireland, Scotland and Wales).

### 2.2 Data collection

#### 2.2.1 Database searches

To identify relevant policy and practice documents, we used a snowballing technique to identify the necessary sources among: Google and Google Scholar; governmental, academic, and health organisation websites; and peer literature and grey literature.

For Google Scholar, the first 10 pages (100 items) were collated. For all other sources, all returned items were collated. One team member (EH) reviewed each literature item for potential relevance to the research questions.

#### 2.2.2 Expert reviewers

We aimed to maximise the accuracy and completeness of our analyses through verification of the country-specific narrative synthesis document by experts in all included countries across two phases between 2021 and 2023. Experts in each country were identified purposively from documents collected during the literature search. There were 1–3 experts per country, depending on availability at the time of invitation. Experts were chosen on the basis of their deep insight into how PEoLC data were collected and utilised in their country through their active involvement in key activities such as policy development, management or oversight of data sets, research, or authorship of relevant articles.

In the first phase, experts were contacted in each country. An email was drafted which stated the aims of the investigation, explained why the individual was being contacted specifically, and requested that they provide written feedback on the country-specific narrative synthesis, reviewing the content for accuracy and comprehensiveness and highlighting any additional relevant evidence not cited. They were also asked to share insights into the next steps for the palliative care data infrastructure in their country. If they were unavailable to provide input on the drafted summary, we asked them to identify or directly contact any colleagues that might be able to assist in the project. Experts received reminders one week after original email contact. During the second phase, each country-specific draft was revised to incorporate this input and the final synthesis was then sent back to the expert for final review and approval.

#### 2.2.3 *Quality* assessment

The policy document review undertaken did not lend itself to the use of a standardised quality assessment tool. We assessed each of the documents for inclusion based on relevance to the aims and objectives of the review and then worked with local experts to check the validity of the data sources identified and the data points extracted.

#### 2.2.4 *Data* extraction

All relevant documents, including webpages were imported into NVivo (version 12), a software programme utilised for qualitative data analysis, and coded for specific data points [10]. Documentation was examined in line with the research question, and relevant data extracted accordingly. For countries with a substantive PEoLC database, the following were documented:

- Description of the database from the perspective of:
  • name
  • type (encompasses the information sources and the level of detail of the information included in the database)
  • year and context for introduction (including relevant policy)
  • purpose
  • population included
  • whether patient level or episode level
  • variables and outcome measures collected
  • ability to link with other national data sources
  • how the database is populated/data generated
  • governance including validation and evaluation
- How the database is used to inform palliative care planning and services
- Documented limitations of the database
- Factors influencing implementation and sustainability of the database
- Considerations to improve the PEoLC data infrastructure

For those countries without a substantive PEoLC database, documents were summarised to describe efforts to improve their PEoLC data infrastructure.

### 2.3 Data analysis

#### 2.3.1 Generating country-specific data using narrative synthesis

Narrative synthesis was employed to review identified material separately for each of the 16 countries. Narrative synthesis collates findings into a coherent textual narrative, grouping and reporting the key findings across data sources. This method is well suited to this form of inquiry, whereby the data collected and processes described are predominantly text-based, and in which depth of detail is important to the audience (in this case, policy-makers). This resulted in a country-level narrative synopsis.

#### 2.3.2 Combining country-specific data using thematic synthesis

Each expert-verified country-level synthesis was imported into NVivo (version 12). We undertook a thematic synthesis of these syntheses. This resulted in a comprehensive description of the similarities and differences in the PEoLC data infrastructure across the countries examined.

#### 2.3.3 Non-English-language materials

The majority of policy documents from non-English-speaking countries had English translations of the documents available. Where documents were identified which required translation to English for data extraction, we used Google Translate. For quality assessment, the expert from each country reviewed the document closely in order to identify any lack of clarification or mistranslation present, and clarified these points. These clarifications were minimal and fully incorporated into the summaries.

## 3 Results

The aim of this research was to synthesise international practice and experience with respect to the use of routine data in PEoLC planning and evaluation, underpinned by the four objectives outlined in Section 1.2.

In this section, we present the key findings of our narrative synthesis for each of these four objectives. Further detailed information on the palliative care data infrastructure within each of the countries examined is presented in Supplementary files 1 and 2. Supplementary file 1 provides a high-level overview of the palliative care data infrastructure in the countries reviewed. Supplementary file 2 provides a synopsis of the considerations locally to improve the palliative care infrastructure in each of the countries reviewed. The full country-level narrative syntheses are available here.

### 3.1 Descriptions of data sources and how they are operationalised

#### 3.1.1 Types of data sources identified

The types of data sources identified are summarised in Table 1. Several countries have established their own bespoke PEoLC databases; others harness existing data sources, and capitalise on the existence of unique patient identifiers to link across existing data sources. Many countries use a combination of data sources to inform and evaluate their palliative care system. While there is a substantive amount of heterogeneity, we grouped identified data sources into five categories:

- patient-level PEoLC registries/databases;
- patient-level registries, not specific to PEoLC;
- palliative care services activity databases, including minimal databases;
- existing administrative databases;
- other sources.

**Table 1:** Categorisation of databases/ data sources used in palliative care.

Type of databases/data sources used	Countries (data source)
Patient-level palliative care registries/databases	Australia (Palliative Care Outcomes Collaboration (PCOC)); Denmark (Danish Palliative Care Database (DPD), '*Dansk Palliativ Database'*); Germany ('*DGP Nationales Hospiz- und Palliativregister'*); Sweden (Swedish Register of Palliative Care (SRPC), *'Svenska palliativregistret'*); Switzerland (SwissPALL)^a^; UK (OACC and RESOLVE^2^, GP Registers)
Patient-level registries, not specific to palliative care	Cancer registries collecting data on palliative care (Norway, Belgium, Canada); Other health and social care registries (Finland)
Palliative care services activity databases, including minimal databases	Austria (Hospice Austria); New Zealand (Hospice NZ); Ireland (MDS); UK (MDS)^3^
Existing administrative data	Belgium; Canada; England; Finland; France; Japan; New Zealand; Scotland; The Netherlands; Wales
Other sources of palliative care data:	
- National surveys	Finland; France; Japan; Switzerland
- Audit	England; Northern Ireland; Wales (NACEL)
- Quality indicator database	Belgium (QPAC)
- interRAI assessments	Belgium, Finland, NZ, Switzerland

a SwissPALL is still under construction.^2^ RESOLVE is working to establish a Palliative Care Outcomes Registry in the UK, based on the OACC suite of outcome measures. ^3^The UK MDS was discontinued in 2017.

#### 3.1.2 Patient-level databases − specific to PEoLC

We identified national patient-level PEoLC databases in six countries: Australia[11,12], Denmark[13,14], Germany[[15], [16], [17], [18]], Sweden[[19], [20], [21]], Switzerland[[22], [23], [24]] and the United Kingdom[[25], [26], [27], [28], [29], [30]]. These differ in their scope, the type of data collected, and the population included (Supplementary Table 1).

#### 3.1.3 Patient-level databases − not specific to PEoLC

Cancer registries in three countries are starting to collect data related to PEoLC: Norway [31], Belgium [32] and Canada [33]. Finland does not have a PEoLC database, but makes significant use of its population and health registries to evaluate palliative care, predominantly in a research capacity [[34], [35], [36], [37], [38], [39]] (Table 1).

#### 3.1.4 Palliative care services activity databases, including minimal databases

Activity databases typically capture data at the service level (aggregate rather than individual data). We identified three activity databases that are currently in use in Austria [40], Ireland [41,42] and New Zealand [43]. Further details are summarised in Supplementary Table 2. These databases are used to provide information on aspects of care for adults who access specialist palliative care services, typically focusing on the structure and processes of care; for example, number of staff, number of admissions and length of stay [26].

#### 3.1.5 Existing administrative data

Routine administrative data are used to provide information on access to and the quality of palliative care services in seven countries [[43], [44], [45], [46], [47], [48], [49], [50], [51], [52], [53], [54], [55], [56], [57], [58], [59], [60], [61], [62], [63], [64], [65], [66], [67]]. Several countries also capitalise on their unique patient health identifiers to link across several administrative databases. See Supplementary Table 3 for further details.

#### 3.1.6 Other sources of PEoLC data

We identified multiple other sources of PEoLC data being collated across the 16 countries and grouped these into four categories: national surveys [48,[68], [69], [70], [71], [72], [73], [74], [75]], quality indicators databases [76], audit [77,78] and comprehensive clinical assessments utilising various interRAI assessment instruments [22,55,69,[79], [80], [81]]). See Supplementary Table 4 for further details.

#### 3.1.7 *Population covered and* content

Details of the populations covered by PEoLC databases and their content are provided in Table 2 [11,13,[19], [20], [21], [22], [23], [24], [25], [26], [27], [28], [29], [30],[40], [41], [42], [43],[57], [58], [59],^68^,[72], [73], [74],[77], [78], [79],[82], [83], [84], [85], [86], [87], [88], [89], [90], [91], [92], [93], [94]] and outcome measures collected in these databases are expanded on in Supplementary Table 5[19,[21], [22], [23], [24],^88^,[95], [96], [97], [98], [99], [100], [101], [102]]. The majority focus on care delivered by specialist palliative services. Notable exceptions that encompass a wider population are found in Sweden, the United Kingdom and the Netherlands. The variation in content reflects differences in health system-level factors such as existing IT infrastructure, health system financing models, and requirements for clinical governance and oversight (See Section 3.2 for further details on how the data infrastructure is utilised).

**Table 2:** Databases/data sources – population covered and content included.^a^

Country (database/data source)	Population covered	Content included
Australia [11,82,83]	Patients in receipt of **SPC services.** This comprises inpatient services, which includes patients seen in designated or non-designated beds under the direct care of the palliative care team, and community services which includes patients seen in the community and through ambulatory (outpatient) clinic episodes. Represents approximately 85 % of all palliative care patients referred to specialist services in Australia	Data are captured at three distinct levels. Patient-level includes demographics such as age, sex, indigenous status, preferred language and country of birth. Episode-level describes the setting of palliative care service provision, and information relating to the facility or organisation that has referred the patient (if a new patient), how an episode starts and ends, level of support at start and end, and, if applicable, place of death. Phase-level information describes the clinical condition of the patient.
Austria (Database of Hospice Austria) [40]	Patients in receipt of **specialist palliative care services** in hospices and other palliative care facilities. Two recent projects of Hospice Austria include the integration of hospice and palliative care in nursing homes and mobile care and support at home	A yearly comprehensive report provides details on: number of specialist hospice and palliative facilities (and beds), reported separately for hospice teams, mobile home palliative care teams, day hospices, hospital palliative care team, inpatient hospices and palliative wards/unit; specifics of patients cared for (number of patients per service type, age, gender, disease, place of care, place of death); staffing of services.
Belgium (QPAC) [76,84,85]	Patients under the care of **specialist palliative care facilities** in the hospital and in the home environment including: specialised unit for palliative care in the hospital; mobile hospital palliative care team; and multidisciplinary team in home care. Two groups of patients are selected: patients who receive care from the palliative care facility at the time of the measurement, and patients who received care but died in the 6 months to 6 weeks before the time of measurement.	QPAC indicators evaluate processes and outcomes of care within eight distinct domains of care including: physical aspect of care; psychosocial aspects of care; information, communication and care planning with the patient; information, communication and care planning with family; information, communication and care planning between care providers; type of care and; circumstances surrounding death; coordination and continuity of care; and care for family.
Denmark (Danish Palliative Care Database – DPD) [13,86]	All patients referred to and / or in contact with **specialist palliative care** (SPC) at hospice and / or in palliative teams / units.	The main variables are data about referral for patients admitted and not admitted to services, type of the first SPC contact, clinical and socio-demographic factors, multidisciplinary conference, and the patient-reported European Organisation for Research and Treatment of Cancer Quality of Life Questionaire-Core-15-Palliative Care questionnaire.
Finland (InterRAI assessments in Nursing homes) [68,79,87]	Data for each resident in nursing homes is collected using admission, every six months, or when conditions change. In 2018, more than half of the municipalities where RAI home care tools were used systematically performed RAI assessments for more than 50 % of their clients over the age of 75 years; and municipalities achieved up to 70 % RAI assessment coverage in home care.	The RAI is a 400 item observational questionnaire filled in by staff. The questions cover the most important aspects of the client's state of health and care. Several well validated scales sets of outcome measures can be derived from the questionnaire including: physical activities of daily living; instrumental activities of daily living; cognitive performance scale; changes in health, end-stage disease and symptoms and signs; body mass index; depression rating scale; pain scale; resource utilisation groups; and quality indicators.
Germany (Nationales Hospiz-und Palliativregister − HOPE) [[88], [89], [90]]	Patients in receipt of **care in palliative and hospice care institutions** including: inpatient palliative care units, inpatient hospices, oncology and geriatric units, specialist outpatient palliative care teams, mobile (SAPV) teams, and nursing services [83,110,111].	Includes an assessment of the Eastern Cooperative Oncology Group performance status. In addition to the core database, modules on various topics and questions are provided: the Minimal Documentation System for self-assessment of the symptoms and the condition of the patient, the German version of the Palliative Outcome Score or the basic psycho-oncological documentation.
Ireland (Minimum Data Set − MDS) [41,42]	Covers the population receiving SPC services including: inpatient units (IPU) (hospices and dedicated palliative care beds in acute hospitals), community services (care provided to people in their normal place of residence by members of SPC team), day care services and SPC data in acute hospitals in acute hospitals (since 2016).	Service-level information includes: total referrals; appropriate referrals; numbers triaged within 1 working day of referral; wait time for SPC; number of discharges, transfers and deaths from the services; number of beds occupied (specialist inpatient palliative care services); activity in SPC bereavement support. Patient-level information includes: age, sex, diagnosis, location prior to and after discharge, place of death.
Japan (Surveys) [22,[72], [73], [74]]	The Japan Hospice and Palliative care Evaluation (J-HOPE) cross-sectional surveys are self-reported anonymous questionnaires filled in by bereaved family members of patients from palliative care units [67,68]. The annual survey which the Japanese Society of Palliative Medicine (JSPM) registry examines structure, processes and outcomes from over 500 palliative care teams.	The J-HOPE surveys evaluate the 'quality of care' and 'quality of life' at Palliative Care Units by utilising the Care Evaluation Scale and Good Death Inventory respectively. The JSPM registry includes outcomes such as: discharge status (i.e., discharged from service; discharged to palliative care units; discharged to other institutions; ended follow-up (alive); continued follow-up; or death.
New Zealand (Database of Hospice New Zealand) [43]	Patients in receipt of in-patient and community palliative care delivered by palliative care hospice services.	Data are collected in October for the previous 12-month period to the end of June. This includes service data, financial data, and community services support data (education and clinical advice and support).
Sweden (Swedish Register of Palliative Care − SRPC) [[19], [20], [21]]	The registry includes the **entire population at end-of-life care** regardless of age, diagnosis, place of death or level of care, as is it a measure of care in the week before death, filled in upon the death of the patient.	Data collection is based on the End of life Questionnaire (ELQ), using the British Geriatrics Society’s statement on what constitutes a good death as an important inspiration and guide. A relative completes a second questionnaire called a ‘related party questionnaire’, after the death has occurred. This contains similar questions as in the ELQ, but also some questions directed to related parties regarding treatment and information.
Switzerland (SwissPALL) [[22], [23], [24]]	Patients in receipt of care at certified **SPC institutions** including mobile teams and specialist long-term care facilities	Structural data will be collected yearly at the service level. Patient data will be collected at entry, exit (or both), including: demographics; diagnosis; palliative care phase; function and symptom (e.g., Barthel Index and the Edmonton Symptom Assessment System), on initial assessment and at exit; treatment provided; and discharge destination.
The Netherlands (Informatiesysteem Palliatieve Zorg) [[57], [58], [59],[91], [92], [93], [94]]	The information creates a 'palliative care population', consisting of individuals who died of cancer, heart disease (chronic), respiratory disease (chronic), stroke, kidney disease (chronic), liver disease (chronic), dementia / senility, neurodegenerative disease and HIV/AIDS.	Data from the linked administrative data sources includes: characteristics of the palliative care population and use of hospital and general practitioner care; acute care in hospitals and at a general practice (GP) out-of-hours service of the palliative care population; prescribing medication by GPs in the palliative care population; and intensive care unit admissions and other potentially inappropriate hospital treatments in the palliative care population.
United Kingdom (The Outcomes and Complexity Collaboration – OACC and the RESOLVE programme) [25,26]	Patients receiving **SPC services** which are across three different settings: hospice and SPC units; hospital advisory support teams in acute hospitals; community care	Consists of a suite of outcome measures including the stage of illness, the patient’s functioning, symptoms and other important concerns, and the impact palliative care services are having on the patient’s and family’s (unpaid caregiver’s) quality of life.
United Kingdom (General Practice Care Registers) [[27], [28], [29], [30]]	**Patients who General Practitioners have identified as those that may benefit from palliative care.** This includes people who may not be in contact with secondary care for specific conditions, but who may have a range of advanced conditions and could be approaching the end of life	There is a lack of documentation on what these registers look like.
United Kingdom (National Audit of Care at the End of Life) [77,78]	The audit focuses on the care delivered during this last admission to hospital prior to death. It includes a population of patients who die in hospital (acute, community hospitals and mental health inpatient facilities), whose death was either recognised or not unexpected.	Examines how hospital care measures up to nationally agreed quality standards in: Recognising the possibility of imminent death; Communication with the dying person; Communication with families and others; Involvement in decision making; Needs of families and others; Individual plan of care/Place of death; Families’ and others’ experience of care; Governance

a Secondary administrative data sources are not included in this table, with the exemption of the Palliative Care Information System in the Netherlands which though is derived from administrative data, is a standalone system. See the country-level profiles of Belgium, Canada, Japan, New Zealand, England, France, Finland, Scotland, and Wales for details on the use of existing administrative data.

Outcome measures are currently collected in national PEoLC databases in Australia [[95], [96], [97], [98]], Denmark [101], Germany [99,102], and Sweden [19]. Additionally, outcome measures are being incorporated in Austria, [99,100] England [88,97,99] and Switzerland [[21], [22], [23], [24]] (Supplementary Table 5).

In most instances, data are reported from the services to the PEoLC databases and data sources utilising a variety of methods, including a combination of paper filling, and web based applications/data entry portals. To date there is no automatic transfer of information from electronic health records to the PEoLC databases. See Supplementary Table 6 for further details on data collection and management arrangements [11,13,14,[21], [22], [23], [24],^31^,^41^,[57], [58], [59],[71], [72], [73],[76], [77], [78], [79], [80],[89], [90], [91], [92], [93], [94],^100^,[103], [104], [105], [106], [107], [108], [109], [110]].

#### 3.1.8 Governance and validation

Table 3 provides an overview of the information available on governance, validation, and evaluation for the established PEoLC databases examined. Data governance is the process of managing the availability, usability, integrity and security of the database/data source. Validation refers to ensuring the integrity (accuracy and clarity) of the data collected. In this instance, it does not refer to validation of outcomes measures collected in the databases (details of validation studies are provided in the country-level synopses in supplementary additional materials).

**Table 3:** Governance, validation and evaluation of the databases.

**Country (database/ data source)**	**Governance**	**Validation**	**Evaluation of the database**^a^
Australia (PCOC)	✓	✓	✓
Austria (Hospice Austria)	✓	✓	
Belgium (QPAC)	✓	✓	✓
Denmark (DPD)	✓	✓	✓
Germany (DGP Nationales Hospiz- und Palliativregister)	✓	✓	✓
Ireland (MDS)	Lack of information	Lack of information	
New Zealand (Hospice NZ)	✓	✓	
Sweden (SPRC)	✓	✓	✓
Switzerland (SwissPALL)	Lack of information	Lack of information	NA
The Netherlands (IPZ)^b^	✓	NA^b^	NA^c^
UK(OACC and RESOLVE)^d^	✓	NA	NA
England, Northern Ireland, Wales (NACEL)	✓	Lack of information	Lack of information

a From the perspective of usefulness of the database or its impact on patient care.

b Secondary use of existing (administrative) data.

c The Information system is new and as such has not been evaluated for its impact on patient care.

d RESOLVE is working to establish a Palliative Care Outcomes Registry in the UK.

Relative to other aspects of the review, there was little information on the governance arrangements across the PEoLC databases (See Supplementary Table 7 for an overview) [116,118]. Similarly, not all countries profiled provide details of how the data in their PEoLC databases/data sources are validated, or the integrity of the data held within. Notable exceptions were Australia [12,83,111], Austria [100,103], Belgium [[112], [113], [114]], Denmark [13,14,115], and Sweden [21,117] (Supplementary Table 7).

### 3.2 How the PEoLC data infrastructure is used in informing palliative care service planning and care delivery

#### 3.2.1 Purpose of databases/data sources

The PEoLC data infrastructure is utilised by various stakeholders within each country. While we identified a range of PEoLC data requirements expressed across the documentation (e.g. commissioning, benchmarking, audit, statutory reporting for registries and research) the purpose of the databases/data sources can be broadly categorised into two domains: (1) examining progress towards the implementation of PEoLC policy, and to inform development of palliative care service; and (2) measuring quality indicators (process, structural, outcomes) and providing feedback and insight to services to improve quality of care (Supplementary Tables 8 and 9) [[119], [120], [121], [122], [123], [124], [125], [126], [127], [128]].

It is worth nothing that most databases and data sources serve more than one purpose. For example in the United Kingdom, the Outcome Assessment and Complexity Collaborative programme is used to inform at the following levels: 1) at the individual level to inform the care of the individual, and the assessment at the start of spell of care identifies the complexity of needs; 2) at the service level to provide aggregated information about residents, to shape and plan services, and for quality assurance; and 3) at the population level for commissioning and research such as measuring and evaluating care and interventions, building knowledge to advance practice, and to demonstrate impact [25,26,128].

Among those examining progress towards policy and development of services, a majority of databases and data sources are used to examine the status of the relevant palliative care system, from the perspective of access to services, and geographical variation in same (Supplementary Table 8).

Among those measuring quality indicators, the majority have developed and apply quality indicators to measure performance of their palliative care sector, either at the national level, or at the individual service level (Supplementary Table 9). Furthermore, the majority of purposefully established PEoLC databases provide individualised feedback to the services − frequently using quality indicators identified above − to allow services benchmark their service against other services [111].

#### 3.2.2 *Evaluation of utility or* impact

Evaluation in this context refers to whether there has been any reported evaluation of the database from the perspective of its perceived usefulness and acceptability within healthcare settings or its impact on the quality of patient care (e.g. process and outcomes). Similar to governance and validation, there was limited information available on the evaluation of these databases/data sources (Table 3). The evidence base about the impact of the databases on process of care or patient outcomes is at an early stage of development, with few studies or external evaluations conducted thus far. However, there were some examples identified from Australia [129], Belgium [112,130], Germany [17], Sweden [131] and the United Kingdom [[132], [133], [134]] (Table 4). Overall, the reported findings suggest that these databases are regarded as tools that improve service delivery and patient outcomes by facilitating benchmarking within and across services, supporting better symptom recognition, and enabling discussions about quality of life.

**Table 4:** Evaluations of database utility or impact.

Countries (database/data source)	Key evaluation findings
Australia (The Palliative Care Outcomes Collaboration − PCOC)	A 2020 report by the PCOC group highlights the improvements achieved by participating services over the span of more than a decade [129]. There has been no external evaluation of the database or its impact on patient care to date.
Belgium (QPAC)	The face validity, feasibility, discriminative power and usefulness of the set of quality indicators was tested using a mixed methods approach [112]. A recently published study, found a large risk-adjusted variation across the quality indicator scores, suggesting that repeated and standardized quality improvement evaluations can allow teams to benchmark themselves to each other to identify areas of their palliative care delivery that need improvement [130]. To the best of the authors’ knowledge, there has been no evaluation of the impact of the database on patient care.
Germany (Nationales Hospiz- und Palliativregister − HOPE)	HOPE is continuously adapted and expanded to meet requirements based on the trends in data collected. While in the first few years there were profound changes not only in the content, but also in the structure of the documentation system, the questionnaire has not changed substantially in the last few years, suggesting that HOPE is now well adapted to the needs of clinical practice [17].
Sweden (Swedish Register of Palliative Care − SRPC)	An evaluative study published in 2012 examined whether participation in the SRPC during a three-year period increased the quality of palliative care with respect to eight pre-determined quality indicators [131]. The study found that registration of a unit in the register was correlated with improved end-of-life care by (i) decreasing the prevalence of six examined symptoms; (ii) increasing the prescription of as needed medications for pain, nausea, anxiety and death rattle, and (iii) increasing the proportion of patients dying in their place of preference and (iv) increasing the proportion of next of kin offered a follow-up appointment after the patient’s death. The authors contributed this improvement to the ability of the units to access their own specific data; the ability to compare their data over time and across similar units; to awareness among staff of where improvements could be made; and to improved recording in the medical notes [131].
United Kingdom The Outcomes and Complexity Collaboration (OACC) and the RESOLVE programme)	Two studies reported that the use of patient-centred outcomes data in palliative care populations has an impact on processes (e.g., better symptom recognition, more discussion of quality of life) and outcomes of care (improved emotional and psychological patient wellbeing [132,133]. This is further reported upon in a review of the international evidence that measurement of indicators of desired outcomes improves the quality of and access to palliative care, in order to apply them to the Canadian context [134]. However, to the best of the authors’ knowledge, there is currently no published evidence of the impact of participation in OACC or RESOLVE on patient care.

### 3.3 Overcoming the gaps and limitations

#### 3.3.1 Gaps and limitations identified

The majority of countries documented the gaps in their PEoLC data infrastructure, and highlighted the limitations of their existing data sources. Our synthesis identified the following gaps and limitations in the existing PEoLC databases/data sources:

- Gaps in the population included in the databases/data sources.
- Lack of patient-reported experience measures and patient-reported outcome measures.
- Uncertainty about the validity and sensitivity of the data and measures/indicators.
- Reliance on clinician-reported versus patient-reported measures.
- Use of existing administrative data is convenient and efficient, but limits the scope of what can be examined.
- Not capturing the trajectory of care within and across services.
- Inability to link the databases with other health care registries.
- Selective or low participation rates among services with respect to data reporting.

In most instances, these gaps and limitations are commonly shared across all five types of PEoLC data sources identified in this review. Detailed description of these themes with examples are provided in Table 5 [[135], [136], [137], [138], [139], [140], [141]].

**Table 5:** Documented limitations of the databases/data sources.

Limitations	Types of databases	Descriptive examples
Gaps in the population included	• patient-level PEoLC registries/databases; • patient-level registries, not specific to PEoLC; • palliative care services activity databases, including minimal databases; • existing administrative databases; • other sources	Firstly, palliative care data are largely concerned with specialist palliative care services, with a lack of data collected from services offering what is often referred to as 'basic palliative care'. Expansion of data collection into these other settings Is recognised as being extremely challenging. Secondly, palliative care data are heavily skewed towards patients with cancer, with substantially less information on other people with life-limiting illnesses. For example, in the Danish Palliative Care Database, the vast majority of patients (91 %) who were registered and who died in 2019 had cancer [14]. Thirdly, there is an absence of data on patients referred to palliative care series but who do not access the care. It appears that these data are only captured by Denmark, where access to specialist palliative care services is judged to be an important indicator of quality [13].
Lack of patient-reported experience measures (PREMS) and patient-reported outcome measures (PROMS)	• patient-level PEoLC registries/databases; • patient-level registries, not specific to PEoLC; • palliative care services activity databases, including minimal databases; • existing administrative databases; • other sources	Activity databases lack patient-level data to inform about the impact of services on the quality of care such as improvements or maintenance of functional status, reductions in symptom severity and alleviation of family burden [26]. Only a few of the data sources examined were able to provide details of patient reported outcome mesuares and/or patient reported experience measures. Fewer still were able to provide details of changes over time in these measures; the Palliative Care Outcomes Collaboration in Australia and Outcomes and Complexity Collaboration in the United Kingdom, being exceptions – with some measures reported directly by patients or proxies. In the Danish Palliative Care Database, data on patient's quality of life is currently only captured at first contact with specialist palliative care, though this is likely to change in the future [14].
Validity and sensitivity of the data and measures/indicators	• patient-level PEoLC registries/databases; • patient-level registries, not specific to PEoLC; • existing administrative databases; • other sources	*External validity* Many countries highlighted that the measures and indicators used have been developed and validated in different patient populations and settings from the ones they are currently being used in. For example, in Sweden, the validity of the ELQ has been examined in specialised palliative healthcare units, mainly with patients with cancer [110,136], and there is a need to validate it in patients dying from diseases such as cardiovascular diseases or dementia [19]. In Belgium, it was originally anticipated that QPAC would also be implemented in nursing homes, but the decision was made not to include these facilities, as the setting appeared to be too different from the specialised palliative care services in terms of organisation and structure of care and in characteristics of the population cared for. Following a pilot in 2019, new indicators on palliative care in nursing homes were added to the regional database for palliative care by the Flemish Institute for Quality of Care [23,137]. *Internal validity* Data collection relies on a large number of clinicians to collect data at the time of clinical service provision, potentially limiting the consistency of data. For example, in Sweden, even though the data reported to the Swedish Register of Palliative Care is based on documentation in medical charts, validity is not absolute, in particular regarding symptom prevalence. A validation study, published in 2017, found that the second iteration of survey questionnaire contained more items with high levels of agreement between registrations in the Swedish Register for Palliative Care and notes in the patients’ medical records, than the first iteration, but some items still had low validity, predominantly items concerning outcomes of care, and most items about symptom occurrence and relief had room for improvement [11]. *Sensitivity of the measures used* Many of the measures that are used to judge success of policy implementation are only proxies for outcomes rather than actual care outcomes. For example in Wales, which measures the percentage of people who receive palliative care based on medical specialty and ICD-10 coding, it is reflected upon that this measure is hardly sensitive enough to capture whether the overall aims of the strategy are being met [138].
Clinician reported versus patient-reported measures lack insight	• patient-level PEoLC registries/databases; • patient-level registries, not specific to PEoLC; • other sources	Several of the documents reviewed highlighted the concerns that when data are clinician-reported and not reported by the patients, there is a lack of insight into the patients’ own experiences of the end-of-life care provided. In Germany, for example, the Nationales Hospiz- und Palliativregister (HOPE) tool (HOPE-SP-CL) is documented by staff, which may differ from self-assessment by patients, as staff assessment has been shown to underestimate the intensity and severity of symptoms such as pain [99]. Hence, the combination of staff and patient self-assessment is preferable, and thus a patient questionnaire (MIDOS) is recommended as a self-assessment instrument in addition to the core HOPE-SP-CL database [89]. In Sweden, it is also recognised that clinician-reported measures result in a lack insight into the patients’ own experiences of the end-of-life care provided [139]. While attempts have been made to capture this somewhat, by the provision of a' related party' survey, the number of registrations of the 'related party' survey has been low compared to the ELQ and has not increased significantly in recent years [117]. A majority of measures included in the Palliative Care Outcomes Collaboration in Australia and Outcomes and Complexity Collaboration in the United Kingdom are clinician-reported.
Use of existing administrative data limits what can be examined	• patient-level registries, not specific to PEoLC; • existing administrative databases; • other sources	There are clear gaps in what aspect of palliative care can be assessed using existing administrative data. For example, it is noted that In the Netherlands, only three of the nine domains of the Quality Framework can be measured using routine care records [140]. Much of the information for criteria from the other domains is not registered in any of the data sources examined. A major challenge to expanding the remit of the information system to include patient reported outcome measures and/or patient reported experience measures to provide this information is that these measures are not uniformly documented across the settings. Furthermore there are no routine care registrations about palliative care in hospice settings available for linking in an integrated information system [57]. In Finland, a weakness identified in the health and social care registries is the lack of outcome data relevant to palliative care assessment, e.g., the functional status of the patient, pain score, dementia score, symptom assessment specific to the needs of palliative care, which are recommended in the latest clinical practice guidelines [36]. Furthermore, there is a lack of data on informal care provided [36].
Lack of visibility of the trajectory of care within and across services	• patient-level PEoLC registries/databases; • patient-level registries, not specific to PEoLC; • palliative care services activity databases, including minimal databases; • existing administrative databases; • other sources	Across the majority of data sources reviewed, it is not possible to view the patient's trajectory of care across and within services. This is particularly an issue with activity databases, which are not designed to identify people seen by more than one specialist service; e.g., the same individual might receive care from inpatient, day care and community services. The database can only estimate the minimum number of people seen by each type of specialist palliative care service. In Ireland, it is most likely that a patient recorded in a specialist inpatient unit will also be recorded by community and/or day care services. This means that the numbers of patients recorded in each service are not mutually exclusive [41]. Finland, despite having a well-established unique health identifier, lacks a shared patient record as a result of the multitude of different health care keepers, often with different electronic health care records. Therefore documentation regarding decisions at the end-of-life do not necessarily follow the patient from one service to another [141].
Inability to link the databases with other health care registries	• patient-level PEoLC registries/databases; • patient-level registries, not specific to PEoLC; • palliative care services activity databases, including minimal databases; • existing administrative databases; • other sources	The lack of a unique health identifier which would facilitate linkage with the database/data source been recognised as being problematic in several countries. In Australia, it is currently not feasible to link data from the Palliative Care Outcomes Collaboration with other healthcare databases in Australia. This issue is being resolved in the next iteration of data collection (version 4) [23]. It has been agreed in principal, that there will be identifiers in version 4 which will facilitate linkage to the rest of the health care system, (via probabilistic linkage), which will greatly enrich its use. In New South Wales, efforts are currently under way to implement and enforce the use of a 'unique identifier' for everyone in the State, which would render it much more feasible to link across the health system, with appropriate ethical approval [23].
Selective or low participation rates among services with respect to data reporting	• patient-level PEoLC registries/databases; • palliative care services activity databases, including minimal databases; • other sources	In Sweden, reporting to the Swedish Register of Palliative Care (SRPC) is not mandatory and not all units report data. Units actively decide to join the SRPC, which also means that they may be more attentive to end-of-life care, thus possibly resulting a more favourable picture of end-of-life care [110], given that registration in SRPC is associated with improved quality of care [132]. Likewise, in Germany, service participation in the Nationales Hospiz- und Palliativregister (HOPE) register is optional and remains low [18]. Consequently, incentives should be introduced to encourage participation, perhaps inserting relevant clauses in palliative care contracts. Finally, since HOPE does not have an immediate effect on the patients' treatment or evaluates the professionals' work, there might be a lack of motivation to complete the assessment thoroughly [16].

#### 3.3.2 *Considerations to improve the PEoLC data* infrastructure

Along with the review of the documents within their written feedback, the expert reviewers were asked to provide insight into the next steps for the PEoLC data infrastructure in their country [142,143]. Our synthesis identified the following relevant factors:

- Enhancing the use (and linkage) of existing databases/data sources.
- Establishing new palliative care database/data sources.
- Expanding data capture to include PEoLC provision outside specialist palliative care settings.
- Enhancing data capture at the service level.
- Capturing and reporting patient-reported experience measures and patient-reported outcome measures.
- Developing validated indicators for PEoLC provision
- Improving the methodology to benchmark services.

These are presented thematically in Supplementary Table 10 and in more detail in country summaries provided in Supplementary File 2.

### 3.4 Implementation and sustainability

#### 3.4.1 Factors influencing implementation and sustainability

Several of the countries have documented the facilitators and challenges seen as influential in the implementation and sustainability of their PEoLC databases/data sources [[144], [145], [146], [147], [148], [149], [150]]. The literature on the factors influencing implementation and sustainability of the databases was dominated by experiences in Australia, Belgium, the United Kingdom; Denmark, and Sweden. There was a notable absence of documentation from other jurisdictions.

Our synthesis identified the following factors influencing implementation:

- Existing national policy and associated financial support for the database.
- Fostering a culture of quality and service improvement within and between services.
- Useful and timely feedback to services.
- Resources to services including: administrative support, training and interpretation of feedback.
- Refinement and improvement of the database/data source.
- Motivation for services to participate.
- Presence of a unique health identifier and a history of using registries.
- Other (including technical capability of the services; stepwise implementation of outcome measures; strong academic and clinical partnerships and trust).

Descriptions of these factors with specific examples are provided in Table 6.

**Table 6:** Factors influencing implementation and sustainability of the databases.

Factors	Description
National policy with financial support for the database	In Australia, the publication of a national framework for palliative care in 2000, and associated funding, has been cited as a critical success factor in the implementation of Palliative Care Outcomes Collaboration [112], as has the introduction of a Standard focusing on quality improvement in the National Palliative Care Standards [144]. In the Netherlands, the quality framework published in 2017, which called for all parties involved in palliative care to strive to deliver a quality service, served as an impetus to design a system to provide information on quality. The projects, which led to the developed of the Information System, were only possible because of the grants and government investment. The future of the Information System is dependent on financial support for the personnel and material costs for the technical-operational implementation of the Information System [57]. In the UK, the Minimum Data Set ceased operating in 2017, citing financial reason among others [145]. Financial support (especially from government) has also been identified as critical to the success of the databases in Austria [22], Belgium[76], and Sweden[21].
Fostering a culture of quality and service improvement within and between services	In Australia, the broad engagement of the clinical community who recognise that the measures are clinically useful and can inform practice and service planning, has been cited as essential to the implementation Palliative Care Outcomes Collaboration [112]. A further pre-requisite is close collaboration between services to participate in benchmarking, and continue to improve outcomes systematically [146]. In Belgium a survey of caregivers anticipated that a negative attitude by caregivers towards quality measurement and a lack of skills, time, and staff were mentioned as barriers to successful implementation. In the UK, those overseeing the Outcomes and Complexity Collaboration (and more recently the RESOLVE programme) have cited the importance that outcome measurement is ‘owned’ and managed by clinicians, and not imposed by those managing or commissioning services [26], and that effective leadership and reinforcement throughout the process of implementation influences the embedding of these measures in routine care [147].
Useful and timely feedback to services	In Australia, a timely feedback loop is identified as key to recruiting services that are currently not participating in the data collection [125]. In the UK, a noted challenge in applying the Outcomes and Complexity Collaboration (and more recently the RESOLVE programme) measures is staff not seeing the measures used to improve care (just collected for the sake of collection), which increases the risk of loss of staff engagement[25,134,148]. Useful and timely feedback has also been identified as an important factor in Germany [16] and Sweden [21].
Resources to services including: administrative support, training and interpretation of feedback	The lack of burden on services has been recognised as being a particular advantage of using existing administrative data, for example the Information System (Zorg) in the Netherlands [140]. The relatively light administrative burden of their databases is recognised as been an important factor in the sustainability of the databases in Belgium [76], Germany [17,105] and Denmark. In the UK, the volume of data to be collected in the Outcomes and Complexity Collaboration database − across spells and phases, and the need for consistence in the capture and reporting of same for the data to be meaningful has been sighted as a challenge to implementation [25,134,148]. Hence, they recommends the stepwise implementation of the outcome measures; starting with the Phase of illness and the Australian Karnofsky Performance Score [26]. In Australia, training to populate and use the Palliative Care Outcomes Collaboration database, at clinician and team-level, has been identified as being instrumental in improving the validity of the recording of the phase of the person's condition [22]. Quality Improvement Facilitators train palliative care services, and work with them to interpret their data and initiate change processes to improve outcomes through focusing on quality of care [112]. Similarly, in Belgium, training and support by the research team to the facilities in the advantages of quality indicators and how to use them as part of clinical practice has been described as 'indispensable' [149]. Support is provided in terms of training, documentation, an extensive manual, a help desk that guarantees an answer within 24 h, and a feedback report with the quality scores at the end of each measurement period [149]. Training and support for services has also been identified as being critically important to the success of the databases, in Denmark [22], Finland [79], Sweden [118] in the UK [25,148].
Refinement and improvement of the database/data source	In Australia, there has also been continuous expansion and refinement of the measures in the Palliative Care Outcomes Collaboration database [82,112]. Refinement of the definition of 'getting into' and importantly 'getting out of a phase', and complemented by facilitator training, has led to a noticeable reduction in the variation in the coding of phases, with more stability in the data [22]. An example of refinement of the Palliative Care Outcomes Collaboration database has been the introduction of a patient identifier within the database, which allows for an examination of persons moving between palliative care services. It is expected that the next iteration will go further and capture several patient identifiers which will enable probabilistic linkage with other Australian health care databases, thereby greatly increasing its utility. In the Netherlands it has been highlighted that the Information System needs to be continuously supplemented in the coming years with data from later years, to reveal trends in the use and quality of palliative care. Furthermore, the Information System needs to remain relevant by possibly supplementing it with data from other data sources [57]. Ongoing work to refine the databases and to ensure the data and feedback to services remains relevant, was also cited as being instrumental in the sustainability of databases in Denmark, Germany[17], and Sweden [22,139]. Finally, in the UK, the decision to cease the Minimum Data Set was influenced partly by financial reasons, but also it was stated that 'to remain relevant, the Minimum Data Set would require extensive review and change' [145].
Motivation for services to participate	Mandatory participation in the database only exists in Denmark and Ireland. In Denmark, registration of patients in Danish Palliative Care Database is mandatory for the specialist palliative care units [13]. In Ireland, specialist palliative care services are obligated to return metrics each month. In Belgium, the link between quality indicators and reimbursement has been considered as a possible facilitator to the implementation of quality indicators in specialised palliative care services, but has not been adopted [149]. The QPAC team list the motivating factors for services to participate as the presence of a need to demonstrate quality of care, to perform improvement actions, and to learn from other caregivers and services in the field. In Sweden, registration of cancer deaths in the register is a national quality indicator for cancer care, hence the majority of cancer deaths are registered in the SPRC [139]. In Australia, the National Palliative Care Standards, have been touted as having a positive influence on the participation of PCOC. There is a standard which focuses specifically on quality improvement, and states that the 'services are expected to participate in benchmarking processes to compare its service delivery over time and/or with external organisations' [144]. In the UK,GP practices are incentivised through the Quality and Outcomes Framework to establish and maintain a Palliative Care Register of all patients in need of palliative care/support irrespective of age [27,29,150].
Presence of a unique health identifier and a history of registries	The presence of a unique patient identifier is listed as an influential factor in Denmark, Sweden, Finland and the Netherlands. While a long history of patient registries, especially quality registries, and their use for service improvement is identified as an enabler to the ongoing sustainability of registries in the Scandinavian countries.
Technical capability of the services	Several countries, have invested significantly in platforms to host the data provided by the services, and to ensure the data collection process is as streamlined as possible. In Australia, the Palliative Care Outcomes Collaboration have cited technical challenges as an aspect to seriously consider. Variation in IT systems and data collection protocols has affected service recruitment. Standardising IT systems is difficult given state jurisdictions and their different requirements for collecting and reporting data [112].
Stepwise implementation of outcome measures	In the UK, the Outcomes and Complexity Collaboration (and more recently the RESOLVE programme) recommend the stepwise implementation of the outcome measures; starting with the Phase of Illness and Australian Karnofsky Perfomance Score [26]. These two are very easy to use and they quickly address some important factors relating to the clinical presentation and performance status of patient. It is important that the data capture for Phase of Illness is working well first [134].The next measures to be introduced are IPOS and Views on care; followed by the Zarit Burden Interview and lastly Barthel Index [26].
Strong academic and clinical partnerships	In Australia, the support provided by the University of Wollongong's Centre for Health Service Development, which provides a national training and analysis unit, and publishes the documentation, has been essential to implementation. In the UK, those overseeing the Outcomes and Complexity Collaboration (and more recently the RESOLVE programme) believe that strong academic and clinical partnerships provide solutions to many challenges faced in implementing outcome measures, and they work closely with clinical teams enrolled in the programme [22,26].
Trust	To protect the data against misuse and (wrong or overextended) comparison as well as to ensure the participation of all services, Hospice Austria has obliged to publish the data only on a national level, and not at the individual service level [22].

The factors presented here are not the result of a systemic review of the literature on this topic. They represent the findings of the documentary review of policy and practice document, supplemented with review of relevant peer review and grey literature.

## 4 Discussion

This review of policy and practice documents was undertaken to critically examine and compare the PEoLC data infrastructure in 16 countries with well-established services. It details current practice with respect to PEoLC data infrastructure; draws together the lessons to be learnt by efforts elsewhere to address recognised limitations in the PEoLC data infrastructure; and the documented factors which have been deemed highly influential in implementing and sustaining existing databases.

### 4.1 Learnings from current limitations and the efforts to improve the data infrastructure elsewhere

A lack of data across the spectrum of PEoLC impedes examination of system-wide palliative care practices, which makes addressing policy gaps and informing service planning and delivery difficult. However, extending data collection into other settings such as primary care and nursing homes is recognised as being extremely challenging. With respect to the later, the QPAC in Belgium has recently been adapted for nursing homes, and the quality indicators added to the regional database for palliative care by the Flemish Institute for Quality of Care. In several countries, participation in the interRAI programme of comprehensive clinical assessment across nursing home settings is mandated, or is soon to be. The importance of ensuring that people with life-limiting conditions other than cancer are represented was also discussed in several of the policy documents reviewed.

There is growing recognition of the limitations of activity databases, and the need to enhance data systems to facilitate the capture of patient-reported outcome measures and/or patient-reported experience measures. Outcome measures, including some patient-reported measures, are currently established in palliative care services in several countries including Australia, Denmark, Germany, and Sweden, and the United Kingdom; with efforts underway in other countries to embed the collection of outcome measures in PEoLC (e.g., Austria, Canada, Switzerland, and Wales). Australia has used the Palliative Care Outcomes Collaborative − a national outcomes and benchmarking programme − to create a national longitudinal database that captures information across a patient’s disease trajectory in specialist palliative care. In the United Kingdom, there has been a significant move to embed outcome measures in PEoLC with the Outcome Assessment and Complexity Collaborative project and more recently, the RESOLVE programme; with the later aiming to create an outcomes registry for PEoLC [26]. This likely reflects the drive in the National Health Service towards outcomes-based health care [103], and the move towards commissioning services based on outcomes, rather than structure and process measures (e.g., 24/7 availability of support), meaning that outcomes now serve an important function in palliative care [26].

There is a significant (and growing) use of exiting administrative data sources to provide intelligence around PEoLC. This is particularly the case in Belgium, Canada, France, Finland, Japan, New Zealand, the Netherlands, and the United Kingdom. While some triangulate different data sources to provide a description of geographical variation in access to palliative care services that is useful for planning services (e.g., Canada, England, and France), others capitalise on the existence of a unique health identifier to link across several data sources (e.g., Belgium, Ontario and Alberta in Canada, Japan, Scotland). This facilitates a more comprehensive description of different aspects of their palliative care system, including the examination of population level indicators. For example in Belgium, researchers have developed and applied indicators of appropriate and inappropriate end-of-life care in people with Alzheimer's disease, cancer or chronic obstructive pulmonary disease, measurable with population-level administrative data [45,46].

Importantly, the review has highlighted limitations of re-using administrative data. For example, in the Netherlands, the project personnel responsible for Informatiesysteem Palliatieve Zorg, highlight that only three of the nine domains of the Quality Framework can be measured using routine care records [140]. In Finland, a weakness identified in using the health and social care registries to measure the quality of palliative care is the lack of outcome data relevant to palliative care assessment, e.g., the functional status of the patient, pain score, dementia score, symptom assessment [36]. Additionally, in these registries there is a lack of data on informal care provided to the patient [36].

Enhancing the data captured in clinical systems is a priority among several of the countries examined. For example in Northern Ireland, work is underway on developing access to data in primary care under the General Practice Information Platform initiative. This will support improved identification of patients with palliative care needs in the community through enhanced access to information on primary care clinical systems [22]. Also in Northern Ireland, a new integrated digital information initiative (ENCOMPASS) will provide a more comprehensive palliative care information dataset within the hospital and community services and for those patients receiving care in the independent hospice sector [22]. In Wales, the electronic Palliative Care Casenote, which logs multidisciplinary meeting outputs, assessments and communications, is due to transition from a stand-alone oncology electronic record system to an all Wales Clinical Portal, and will allow greater access to healthcare professionals across all care settings. It will integrate within the National Data Record for Wales which will facilitate data sharing and capture of outcomes [22].

Other aspects that are receiving high levels of attention in the countries examined include: efforts to improve data capture at the service level (e.g., Belgium, Denmark, Sweden); efforts to improve the methodologies that are used to benchmark services (e.g., Belgium and the United Kingdom); and efforts to develop indicators for palliative care. For example in Austria, it is expected that when patient reported outcome measures (e.g. for pain and shortness of breath) are sufficiently established, this will feed into reporting for indicators [22].

### 4.2 Learnings from other jurisdictions with regard to factors which influence implementation and sustainability of established PEoLC data sources

National policies, standards and frameworks are essential to set the direction for the country's data infrastructure, and must be accompanied by the appropriate funding to implement and maintain these data systems.

Within the services, clinician leadership is crucial, as is clinician engagement to foster a culture of quality improvement within the services. Measurements taken need to be valued by all staff, and seen as being clinically useful and applicable to informing practice and service planning, and not seen as collecting data for the sake of it. Otherwise there is a risk of loss of staff engagement. Furthermore, there needs to be recognition by staff of the benefits of benchmarking and the collaboration of peer services to facilitate benchmarking of services.

Feedback to services using their data has to be timely and relevant so that it can be used to inform patient care and service improvement, and evaluate any improvements made after a change in clinical practice. Training of clinicians and teams to improve the quality of data produced, and how to use this data to enhance clinical practice and quality of care in their own service, is a crucial factor. In the Palliative Care Outcomes Collaboration in Australia for example, Quality Improvement Facilitators have responsibility for recruiting and training specialist palliative care service providers, and for working with them to optimise local processes using their own data [22,112].

The administrative load on staff cannot be overly burdensome. In many countries, a significant amount of administrative support is provided to services, from the perspective of data collection, feedback and interpretation of same, to reduce this burden and enhance the use of the data in local decision making.

Strong academic and clinical partnerships can provide solutions to many challenges faced in implementing outcome measures. Furthermore, analytical support can be provided by research institutions through theses academic partnerships.

The introduction of outcome measures into a service should ideally be done in a stepwise manner so not as to overload staff. Lessons can be learnt from the Outcomes and Complexity Collaboration (and more recently RESOLVE) in England, where they recommend the stepwise implementation of the outcome measures; starting with the Phase of illness and Australian Karnofsky Performance Score [26]. This is because these two measures are very easy to use and they quickly address some important factors relating to the clinical presentation and performance status of patient. It is important that the data capture for Phase of Illness is working well first [134].The next measures to be introduced are Integrated Palliative Outcome Score and Views on Care; followed by the Zarit Burden Interview and lastly Barthel Index [26].

The availability (and adoption) of a unique patient identifier is essential so that the database/data source can be utilised appropriately to correctly assess a patients' trajectory of care. Valuable lessons can be learnt from the efforts undertaken by the Palliative Care Outcomes Collaboration in Australia, with respect to this.

### 4.3 Future considerations

Data infrastructure is foundational to health systems, providing the information needed to support core functions such as resource management, service planning, and monitoring delivery and outcomes. While there are many examples of innovative data collection and use practices across these 16 countries, the scope for data to inform decision-making remains limited. Drawing on the MEASURE Evaluation Data Demand and Use conceptual framework [151]), there are clear steps that will help take the field of PEoLC data forward. The framework comprises four elements: data demand, data collection, data availability and access, data use. The elements are highly interdependent and will likely all influence each other.

#### 4.3.1 *Data* demand

Efforts to collect data relevant to PEoLC policy and practice need to be closely align with decision makers’ needs and priorities, addressing the key functions and questions they have. These will vary across settings, depending on how services are organised, funded and delivered, highlighting the importance of ongoing interaction and engagement between data users and data producers. The findings of this review demonstrate that there is significant unmet demand for data about PEoLC outside specialist settings as well as outcomes and experiences across the care trajectory. Future work to strengthen data infrastructure should address these domains, and any other national priorities, to further stimulate data demand. International experience also demonstrates that specifying data requirements and resourcing in national policies, standards and frameworks are all essential to set out a clear direction for the country's data infrastructure.

#### 4.3.2 *Data* collection

The methods and tools used to collect PEoLC data and address information gaps continue to evolve. This review demonstrates that the processes are often resource-intensive, with few examples of automation to date. This approach typically relies on healthcare staff, and in some instances close relations, to capture and record data about PEoLC at the individual- or service-level. This requires ongoing training and funding to ensure that all staff have the skills and knowledge required to collect data in a consistent manner. Activities aimed at exploiting secondary data sources to build the PEoLC data infrastructure should also intensify, with stakeholders collaborating to identify opportunities for linking data sources. Additionally, one of the most cited barriers to developing PEoLC data infrastructure is the lack of validated measures designed to capture data related to the processes and outcomes of interest. Addressing this gap will be central to improving data collection, and is also likely to drive further demand for data. Finally, for those with governance of the PEoLC databases, it is important to have a clear process of continuous refinement of the database in terms of what data are collected, aiming to maximise relevance.

#### 4.3.3 *Data availability and* access

Disseminating data and information widely is crucial for enabling data-informed decision making. There are strong examples of these activities identified throughout this review, including Registry data made accessible online to the general public; benchmarking and activity data analysed and shared with service providers; and the production of ‘Atlas’ reports that collate various data sources related to PEoLC provision and outcomes. However, we also found that the structures for governance and oversight of PEoLC data are often poorly documented and difficult to determine. In order to improve access to PEoLC data, entities responsible for collection and managing data should ensure they have transparent processes for making these available, where possible, to external parties.

#### 4.3.4 *Data* use

Data use is the final phase, where data are translated into practice and policy. Overall, the evidence base about the impact of the PEoLC data infrastructure on service provision or patient outcomes remains very limited. One likely cause is that the metrics being collected do not fully reflect the decision-making needs of all stakeholders. For example, some minimum datasets have been developed to meet commissioning requirements – satisfying the needs of funders, but providing little opportunity for service providers and researchers to use routine data to monitor important issues such as the quality of services, care trajectories near the end of life, and informal care provision. The results of this review underscore the importance of both multidisciplinary collaborations and building data leadership capacity (e.g. clinical champions and data facilitators) in order to foster opportunities for data usage.

### 4.4 Strengths and limitations

It is important to highlight that while our comprehensive international policy review of routine data collection and reporting in PEoLC provides rich data and information, it has two important limitations.

First, we were dependent on compiling data from documents that were in the public domain. It is probable that we missed documents that were not readily available. It is also probable that our searches missed relevant papers as we did not apply the same search terms to each country, instead utilising phrases in the country’s main language(s). The inclusion of local expert reviewers with intimate knowledge of their countries PEoLC data infrastructure is likely to have mitigated this however; and was crucial in verifying the accuracy of data extracted from translated documents.

Second, despite best efforts, we did not succeed in getting an expert reviewer to verify our synopsis of the palliative care data infrastructure in two countries. For the other countries reviewed, we found the interaction of expert reviewers with expertise in their local palliative care data infrastructure highly rewarding, and we greatly valued their significant input into the review.

## 5 Conclusion

We conducted a systematic, wide-ranging review of policy documents to identify international best practice in the collection and use of routine data in PEoLC. Surveying 16 high-income countries with well-established palliative care services, we described the PEoLC data infrastructure in the selected countries, we documented how these databases are used in palliative care service planning and care delivery, we detailed the limitations of existing infrastructure and charted current efforts to address these limitations, and we identified the factors that influence the implementation and sustainability of databases. This first-of-its-kind analysis was conducted to inform explicitly ongoing palliative care policy reforms in Ireland and can also inform other countries’ efforts to improve their PEoLC data infrastructure and palliative care services to meet growing population health needs.

## Consent for publication

Not applicable.

## Ethics approval and consent to participate

Not applicable. No experiments or human participants were involved in this study. Additionally, this study and the manuscript do not report on or involve the use of any animal or human data or tissue.

## Funding

This work is part of a project titled 'Palliative and end-of-life care data in Ireland: establishing the state of the nation, mapping future direction', which is funded through the Irish Health Research Board: 2019/SDAP/012 [PI: May]. For more details on the project aims and outputs, see https://pelci.ie/. The funding body had no involvement with the study design; in the collection, analysis and interpretation of data; in the writing of this manuscript; or in the decision to submit this article for publication. This work was funded by the Health Research Board in Ireland (#SDAP/2019/012).

The funder had no influence over the research questions, analysis, interpretation or reporting. The funding line is designed to address the documented evidence needs of a ‘Knowledge User’ in the Irish health and social care system; that is, someone “in a position of authority to influence and/or make decisions about health policy or the delivery of services and can act to ensure that the findings of the research will be translated to influence decision making and change within their (or other) organisations.”[7] The ‘Knowledge User’ in this project was the palliative care integration and planning programme within the Health Service Executive, the publicly funded healthcare system in the Republic of Ireland. Our research questions were shaped in collaboration with this ‘Knowledge User’, who had no influence over the analysis, interpretation or reporting.

## CRediT authorship contribution statement

**Eimir Hurley:** Writing – review & editing, Writing – original draft, Validation, Investigation. **Peter May:** Writing – review & editing, Project administration, Methodology, Funding acquisition, Conceptualization. **Soraya Matthews:** Writing – review & editing, Writing – original draft. **Charles Normand:** Writing – review & editing, Methodology, Funding acquisition, Conceptualization. **Bridget M. Johnston:** Writing – review & editing, Writing – original draft, Validation, Supervision, Project administration, Methodology, Funding acquisition, Conceptualization.

## Declaration of competing interest

The authors declare that they have no known competing financial interests or personal relationships that could have appeared to influence the work reported in this paper.

## Data Availability

The datasets generated during the current study are available in the Open Science Framework repository, [https://doi.org/10.17605/OSF.IO/PZWME].

## References

[1] Institute of Medicine Committee on Regional Health Data Networks, Health Data in the Information Age: Use, Disclosure, and Privacy, ed. M.S. Donaldson and K.N. Lohr. 1994, Washington (DC): National Academies Press (US). 25144051

[2] Andreu-Perez J. (2015). Big data for health. IEEE J Biomed Health Inform.

[3] Storick V. (2019). Improving palliative and end-of-life care with machine learning and routine data: a rapid review. HRB Open Res.

[4] Morin L., Onwuteaka-Philipsen B.D. (2021). The promise of big data for palliative and end-of-life care research. Palliat Med.

[5] Higginson I.J. (2013). Evaluating complex interventions in End of Life Care: the MORECare Statement on good practice generated by a synthesis of transparent expert consultations and systematic reviews. BMC Med.

[6] World Health Organization, Palliative Care.

[7] Sleeman K.E. (2019). The escalating global burden of serious health-related suffering: projections to 2060 by world regions, age groups, and health conditions. Lancet Glob Health.

[8] (ed.), C.S.R., Global Atlas of Palliative Care, 2nd edition, W.P.C. Alliance, Editor. 2020: London.

[9] Unit., T.E.I., The 2015 Quality of Death Index Ranking palliative care across the world. 2015: London.

[10] Ltd, Q.I.P., NVivo. 2020.

[11] Australian Health Services Research Institute. Palliative Care Outcomes Collaboration: Research and data. 12 November 2020]; Available from: https://www.uow.edu.au/ahsri/pcoc/research-data/.

[12] Allingham, S., Burns, S, Foskett, L, Clapham, S, Daveson, B, Eagar, K and PCOC, Patient Outcomes in Palliative Care in Australia: National report for July – December 2019, Palliative Care Outcomes Collaboration, Editor. 2020, Australian Health Services Research Institute, University of Wollongong.

[13] Groenvold M., Adsersen M., Hansen M. (2016). Danish Palliative Care Database. Clin Epidemiol.

[14] Hansen, M.A., M., Grønvold, M., Dansk Palliativ Database. Årsrapport 2019,. 2020, DMCG-PAL, 2020.: København.

[15] Hofmann S., Hess S., Klein C., Lindena G., Radbruch L., Ostgathe C. (2017). Patients in palliative care. Development of a predictive model for anxiety using routine data. PLoS One.

[16] Hess S., Stiel S., Hofmann S., Klein C., Lindena G., Ostgathe C. (2014). Trends in specialized palliative care for non-cancer patients in Germany–data from the national hospice and palliative care evaluation (HOPE). Eur J Intern Med.

[17] Radbruch, L., Nauck, F., Patientenregister als Forschungsinstrument – am Beispiel der Hospiz- und PalliativErhebung (HOPE). Unknown year.

[18] Leopoldina (Nationale Akademie der Wissenschaften), Palliative care in Germany.Perspectives for practice and research, Leopoldina, Editor. 2015: Köthen (Anhalt).

[19] Martinsson L. (2015).

[20] Andersson S., Årestedt K., Lindqvist O., Fürst C.J., Brännström M. (2018). Factors Associated With Symptom Relief in End-of-Life Care in Residential Care Homes: A National Register-Based Study. J Pain Symptom Manage.

[21] Lundström S., Axelsson B., Heedman P.A., Fransson G., Fürst C.J. (2012). Developing a national quality register in end-of-life care: the Swedish experience. Palliat Med.

[22] Personal Communication with Expert Reviewer. 2021-2023.

[23] Pautex, S., Eychmüller, S., Developing a national database, in Palliative Care Research. Final report on the SAMS funding programme 2014 – 2018. 2020, Swiss Academy of Medical Science (SAMS): Bern, Switzerland. p. 6.

[24] Pautex, S., Eychmüller, S., Project title: SwissPALL 1.0: A national palliative care database: A tool to better define the needs of palliative care patients in Switzerland. Extended summary report. Unknown.

[25] Murtagh, F., Everyone’s talking about outcomes. Presentation. Unknown, Cicely Saunders Institute, Department of Palliative Care, Policy & Rehabilitation, King’s College London: London, UK.

[26] Witt, J., de Wolf-Linder, S., Dawkins, M., Daveson, BA., Higginson, IJ., Murtagh, FEM. on behalf of the Outcome Assessment and Complexity Collaborative (OACC) Team., Introducing the Outcome Assessment and Complexity Collaborative (OACC) Suite of Measures A Brief Introduction - Version 2. 2015, Department of Palliative Care, Policy and Rehabilitation,Cicely Saunders Institute, King’s College London.

[27] NHS England and NHS Improvement, 2020/21 General Medical Services (GMS) contract Quality and Outcomes Framework (QOF). Guidance for GMS contract 2020/21 in England. 2020, NHS: London, UK.

[28] Quality Assurance and Improvement Framework, Guidance for the GMS Contract Wales 2019/20. 2019.

[29] Department of Health. About the Quality and Outcomes Framework (QOF). 19 March 2021]; Available from: https://www.health-ni.gov.uk/articles/about-quality-and-outcomes-framework-qof.

[30] The Scottish Government, NHS Circular: PCA(M)(2019)06. The Primary Medical Services Directed Enhanced Services (SCOTLAND) 2019 Palliative Care Scheme, in Population Health Directorate. Primary Care Division. 2019, The Scottish Government,: Edinburgh, Scotland.

[31] Cancer Registry of Norway. Clinical Registries. Available from: https://www.kreftregisteret.no/en/The-Registries/Clinical-Registries/.

[32] Devos, C., Cordon, A, Lefèvre, M, Obyn, C, Renard, F, et al., Performance of the Belgian health system – report 2019. 2019, Belgian Health Care Knowledge Centre (KCE). Brussels.

[33] Howell D., Rosberger Z., Mayer C., Faria R., Hamel M., Snider A. (2020). Personalized symptom management: a quality improvement collaborative for implementation of patient reported outcomes (PROs) in ‘real-world’ oncology multisite practices. J Patient-Reported Outcomes.

[34] Forma L., Rissanen P., Aaltonen M., Raitanen J., Jylhä M. (2009). Age and closeness of death as determinants of health and social care utilization: a case-control study. Eur J Public Health.

[35] Forma L., Rissanen P., Noro A., Raitanen J., Jylhä M. (2007). Health and social service use among old people in the last 2 years of life. Eur J Ageing.

[36] Aaltonen M., Forma L., Rissanen P., Raitanen J., Jylhä M. (2010). Transitions in health and social service system at the end of life. Eur J Ageing.

[37] Aaltonen M., Forma L., Pulkki J., Raitanen J., Rissanen P., Jylha M. (2017). Changes in older people's care profiles during the last 2 years of life, 1996-1998 and 2011-2013: a retrospective nationwide study in Finland. BMJ Open.

[38] Finnish institute for health and welfare. Care Register for Health Care. Register description. 10 December 2020]; Available from: https://thl.fi/en/web/thlfi-en/statistics/information-on-statistics/register-descriptions/care-register-for-health-care.

[39] Maetens A., De Schreye R., Faes K., Houttekier D., Deliens L., Gielen B. (2016). Using linked administrative and disease-specific databases to study end-of-life care on a population level. BMC Palliat Care.

[40] Hospiz Österreich, Hospiz- und Palliative Care in Österreich 2019. Datenbericht der spezialisierten Hospiz- undund Palliativeinrichtungen, der Bildungsarbeit sowie der Projekte in der Grundversorgung [Data report of the specialized hospice and palliative care facilities for adults and children]. 2020, Dachverband Hospiz Österreich: Wien.

[41] Weafer, J., Toft, S., for the Health Services Executive (HSE) and The Irish Hospice Foundation, National summary of patient activity data for adult specialist palliative care services in the Republic of Ireland, 2012-2015. 2017, The Irish Hospice Foundation: Dublin, Ireland.

[42] Matthews, S., Pierce, M., O'Brien Green, S., Hurley, E., Johnston, BM., Normand, C., et al., Dying and death in Ireland: what do we routinely measure, how can we improve? 2021, Irish Hospice Foundation: Dublin, Ireland.

[43] Hospice NZ. The Year in Review 2019-2020. 20 December 2020]; Available from: https://www.hospice.org.nz/about-hospice-nz/the-year-in-review-2019-2020/.

[44] Maetens A., Beernaert K., Deliens L., Gielen B., Cohen J. (2019). Who finds the road to palliative home care support? A nationwide analysis on the use of supportive measures for palliative home care using linked administrative databases. PLoS One.

[45] De Schreye R., Houttekier D., Deliens L., Cohen J. (2017). Developing indicators of appropriate and inappropriate end-of-life care in people with Alzheimer's disease, cancer or chronic obstructive pulmonary disease for population-level administrative databases: A RAND/UCLA appropriateness study. Palliat Med.

[46] De Schreye R., Smets T., Annemans L., Deliens L., Gielen B., De Gendt C. (2017). Applying Quality Indicators For Administrative Databases To Evaluate End-Of-Life Care For Cancer Patients In Belgium. Health Aff (Millwood).

[47] Canadian Institute for Health Information, Access to Palliative Care in Canada. 2018, CIHI: Ottawa, Ontario, Canada.

[48] Cousin, F., Gonçalves, T., Atlas des soins palliatifs et de la fin de vie en France. Deuxieme edition [Atlas of palliative and end-of-life care in France. Second edition]. 2020, Centre national des soins palliatifs et de la fin de vie (CNSPFV): Paris, France.

[49] Centre national des soins palliatifs et de la fin de vie (CNSPFV). Atlas of palliative and end-of-life care in France - 2nd edition. 2020 26 February 2021]; Available from: https://www.parlons-fin-de-vie.fr/qui-sommes-nous/atlas-des-soins-palliatifs-et-de-la-fin-de-vie/.

[50] Centre national des soins palliatifs et de la fin de vie (CNSPFV). The Center and its missions. The National Center for Palliative and End-of-Life Care is a public body, open to all, a “common house” in which everyone can learn about palliative care and end-of-life. 2021 26 February 2021]; Available from: https://www.parlons-fin-de-vie.fr/qui-sommes-nous/le-centre-et-ses-missions/.

[51] Sato Y., Miyashita M., Sato K., Fujimori K., Ishikawa K.B., Horiguchi H., Fushimi K., Ishioka C. (2018). End-of-life care for cancer patients in Japanese acute care hospitals: A nationwide retrospective administrative database survey. Jpn J Clin Oncol.

[52] Sato Y., Fujimori K., Ishikawa K.B., Sato K., Ishioka C., Miyashita M. (2016). A Preliminary Survey to Measure the Quality Indicators of End-of-life Cancer Care Using the Japanese National Database [Japanese]. Palliative Care Research.

[53] Abe K. (2020). Place of death associated with types of long-term care services near the end-of-life for home-dwelling older people in Japan: a pooled cross-sectional study. BMC Palliat Care.

[54] Morioka N., Tomio J., Seto T., Yumoto Y., Ogata Y., Kobayashi Y. (2018). Association between local-level resources for home care and home deaths: A nationwide spatial analysis in Japan. PLoS One.

[55] McLeod, H., Atkinson, J., Technical Note on Trajectories of Care at the End of Life Research, University of Auckland and University of Otago, Editor. 2019.

[56] Mcleod, H., Atkinson, J., Policy Brief on Trajectories of Care at the End of Life in New Zealand, University of Auckland and University of Otago, Editor. 2019.

[57] Francke A., Oosterveld-Veld M., Boddaert M., Engels Y., van der Heide A., Heins M. (2020).

[58] ZonMw. Towards a system to provide insight into the quality of palliative care. 1 February 2021]; Available from: https://www.zonmw.nl/nl/over-zonmw/onderwijs/programmas/project-detail/palliantie-meer-dan-zorg/op-weg-naar-een-systeem-om-kwaliteit-van-palliatieve-zorg-inzichtelijk-te-maken/.

[59] ZonMw. More insight into (quality of) palliative care by linking existing registration data on hospital care, general practitioner care and causes of death. 1 February 2021]; Available from: https://www.zonmw.nl/nl/over-zonmw/onderwijs/programmas/project-detail/palliantie-meer-dan-zorg/meer-inzicht-in-kwaliteit-van-palliatieve-zorg-door-koppeling-van-bestaande-registratiegegevens-ov/.

[60] National End of Life Care Intelligence Network (NEoLCIN), Atlas of variation for palliative and end of life care in England. Reducing unwarranted variation to improve health outcomes and value. 2018, Public Health England: London, UK.

[61] Public Health England (PHE). Emergency admissions in the 3 months before death. 3 March 2021]; Available from: https://www.gov.uk/government/publications/emergency-admissions-in-the-3-months-before-death/emergency-admissions-in-the-3-months-before-death.

[62] Public Health England (PHE). Older people’s hospital admissions in the last year of life. 3 March 2021]; Available from: https://www.gov.uk/government/publications/older-peoples-hospital-admissions-in-the-last-year-of-life.

[63] Public Health England (PHE). Palliative and End of Life Care Profiles. 3 March 2021]; Available from: https://www.gov.uk/government/collections/palliative-and-end-of-life-care#palliative-and-end-of-life-care-profiles.

[64] Public Health Scotland, Percentage of End of Life Spent at Home or in a Community Setting Financial years ending 31 March 2011 to 2020. An Official Statistics Publication. 2020, Public Health Scotland: Edinburgh, Scotland.

[65] Public Health Scotland Data and intelligence (Previously ISD Scotland). Source Platform. 2021 11 March 2021]; Available from: https://beta.isdscotland.org/topics/source-platform/.

[66] The Scottish Government, Strategic Commissioning of Palliative and End of Life Care by Integration Authorities. 2018, The Scottish Government: Edinburgh, Scotland.

[67] The Welsh Government, Together for Health. End of Life Care Annual Report 2016.

[68] Kalliomaa-Puha, L., Kangas, O. , European Social Policy Network (ESPN)Thematic Report on Challenges in long-term care: Finland, E. Commission, Editor. 2018: Brussels, Belgium.

[69] Tiina Saarto ja asiantuntijatyöryhmä [expert working group], Sosiaali- ja terveysministeriön raportteja ja muistioita 2017:44 Palliatiivisen hoidon ja saattohoidon järjestäminen [Arranging palliative care and convalescent care]. 2017, Social- och hälsovårdsministeriet: Helsinki, Finland.

[70] Social- och hälsovårdsministeriet, Alueellinen kartoitus ja ehdotuksia laadun ja saatavuuden parantamiseksi Palliatiivisen hoidon ja saattohoidon tila Suomessa [Regional mapping and suggestions for quality and to improve accessibility. Status of palliative care and convalescent care In Finland]. 2019, Social- och hälsovårdsministeriet Helsinki, Finland.

[71] Centre national des soins palliatifs et de la fin de vie (CNSPFV). First national survey on structures and human resources in palliative care. Press Relsease. 2020 26 February 2021]; Available from: https://www.parlons-fin-de-vie.fr/press/premiere-enquete-nationale-sur-les-structures-et-ressources-humaines-en-soins-palliatifs/.

[72] Miyashita M. (2015). A nationwide survey of quality of end-of-life cancer care in designated cancer centers, inpatient palliative care units, and home hospices in Japan: the J-HOPE study. J Pain Symptom Manage.

[73] Masukawa K. (2018). The Japan hospice and palliative evaluation study 4: a cross-sectional questionnaire survey. BMC Palliat Care.

[74] Mori M., Morita T. (2016). Advances in Hospice and Palliative Care in Japan: A Review Paper. J Hospice Palliative Care.

[75] Liechti, L., Künzi, K., Stand und Umsetzung von Palliative Care in den Kantonen Ergebnisse der Befragung der Kantone und Sektionen von palliative ch . 2018 Schlussbericht [Status and implementation of palliative care in the cantons. Results of the survey of the cantons and sections of palliative ch 2018 Final report], Bundesamts für Gesundheit (BAG); Direktionsbereich Gesundheitspolitik; Sektion Nationale Gesundheitspolitik, Editor. 2019, Bundesamt für Gesundheit (BAG): Bern, Switzerland.

[76] End-of-Life Care Research Group, Kwaliteitsindicatoren voor palliatieve zorg. Waar zijn we en wat is de toekomst? Beleidsrapport [Quality indicators for palliative care. Where are we and what is the future? Policy report]. 2017, End-of Life Care Research Group (VUB and UGent): Flanders, Belgium.

[77] NHS Benchmarking Network (2020).

[78] NHS Benchmarking Network (2020).

[79] Finne-Soveri H., Hammar T., Noro A. (2010). Measuring the quality of longterm institutional care in Finland. Eurohealth.

[80] InterRAI New Zealand, Interpreting the results of an interRAI Palliative Care assessment, InterRAI New Zealand, Editor. 2017.

[81] Eicher S., Theill N., Geschwindner H., Moor C., Wettstein A., Bieri-Brüning G. (2016). The last phase of life with dementia in Swiss nursing homes: The study protocol of the longitudinal and prospective ZULIDAD study. BMC Palliat Care.

[82] Currow, D.C. The Australian experience of implementing palliative care outcome measures. 2017 10 November 2020]; Available from: http://www.professionalpalliativehub.com/sites/default/files/Outcome%20Measurement%20Event%20Prof%20David%20Currow.pdf.

[83] Allingham S for the Palliative Care Outcomes Collaboration, Palliative Care Outcomes Collaboratio: Data Dictionary and Technical Guidelines Document version 1.2.0. 2012, Australian Health Services Research Institute (AHSRI): University of Wollongong, Australia.

[84] End-Of-Life Care Research Group. More information about quality indicators. 21 January 2021]; Available from: https://www.endoflifecare.be/nl/meer-informatie-over-kwaliteitsindicatoren.

[85] End-Of-Life Care Research Group. Quality indicators for palliative care in Flanders. 21 January 2021]; Available from: https://www.endoflifecare.be/nl/kwaliteitsindicatoren.

[86] Copenhagen HealthTech Cluster. Danish Palliative Care Database. 2020 19 November 2020]; Available from: https://www.danishhealthdata.com/find-health-data/Dansk-Palliativ-Database.

[87] Terveyden ja hyvinvoinnin laitos [Finnish institute for health and welfare]. About the RAI system. 10 December 2020]; Available from: https://thl.fi/fi/web/ikaantyminen/palvelutarpeiden-arviointi-rai-jarjestelmalla/tietoa-rai-jarjestelmasta.

[88] Stiel S., Pollok A., Elsner F., Lindena G., Ostgathe C., Nauck F. (2012). Validation of the Symptom and Problem Checklist of the German Hospice and Palliative Care Evaluation (HOPE). J Pain Symptom Manage.

[89] MASSNAHME ZUR QUALITÄTSSICHERUNG. Darstellung des Projektes, HOPE Hospiz- und Palliativ- Erhebung DOKUMENTATIONS PHASE 2011 Stand 18.2.2011. 2011.

[90] Simon S.T., Altfelder N., Alt-Epping B., Bausewein C., Weingärtner V., Voltz R. (2014). Is breathlessness what the professional says it is? Analysis of patient and professionals' assessments from a German nationwide register. Support Care Cancer.

[91] Oosterveld, M., Reyners, A., Heins, M., Boddaert, M., Engels, Y., van der Heide, A., et al., Factsheet 1: Kenmerken van de populatie en gebruik van ziekenhuis- en huisartsenzorg. 2020, NIVEL/ZonMw/PalZon.

[92] Oosterveld, M., Reyners, A., Heins, M., Boddaert, M., Engels, Y., van der Heide, A., et al., Factsheet 2: Acute zorg in het ziekenhuis en van de huisartsenpost. 2020, NIVEL/ZonMw/PalZon.

[93] Oosterveld, M., Reyners, A., Heins, M., Boddaert, M., Engels, Y., van der Heide, A., et al., Factsheet 3: Voorschrijven van medicatie door de huisarts. 2020, NIVEL/ZonMw/PalZon.

[94] Oosterveld, M., Reyners, A., Heins, M., Boddaert, M., Engels, Y., van der Heide, A., et al., Factsheet 4: IC opnamen en andere potentieel niet-passende behandelingen in het ziekenhuis. 2020, NIVEL/ZonMw/PalZon.

[95] Masso M. (2016). Palliative Care Problem Severity Score: Reliability and acceptability in a national study. Palliat Med.

[96] Aoun S.M. (2011). Measuring symptom distress in palliative care: psychometric properties of the Symptom Assessment Scale (SAS). J Palliat Med.

[97] Abernethy A., Shelby-James T., Fazekas B.S., Woods D., Currow D.C. (2005). The Australian modified Karnofsky Performance Status (AKPS) scale: a revised scale for contemporary palliative care clinical practice. BMC Palliat Care.

[98] Morgan, D.B., A; Cerdor, P; Currow, D., Does Resource Utilization Group-Activities of Daily Living Help Us Better Interpret Australian Karnofsky-Modified Performance Scale? Journal of Palliative Medicine., 2020(Does Resource Utilization Group-Activities of Daily Living Help Us Better Interpret Australian Karnofsky-Modified Performance Scale?): p. 1153-1154. 10.1089/jpm.2020.0163 32877287

[99] Murtagh F., Ramsenthaler C., Firth A., Groeneveld E., Lovell N., Simon S. (2019). A brief, patient- and proxy-reported outcome measure in advanced illness: Validity, reliability and responsiveness of the Integrated Palliative care Outcome Scale (IPOS). Palliat Med.

[100] Zielsteuerung-Gesundheit on behalf of Bundesministerium für Soziales Gesundheit Pflege und Konsumentenschutz, Outcome-Messung im Gesundheitswesen basierend auf dem Mess- und Vergleichskonzept. Detailanalyse relevanter Outcomes im Gesundheitswesen. Aktualisierte Fassung 2020. 2020, Bundesministerium für Soziales, Gesundheit, Pflege und Konsumentenschutz Gesch.

[101] Zielsteuerung-Gesundheit on behalf of Bundesministerium für Soziales Gesundheit Pflege und Konsumentenschutz, Outcome-Messung im Gesundheitswesen basierend auf dem Mess- und Vergleichskonzept. Detailanalyse relevanter Outcomes im Gesundheitswesen. Aktualisierte Fassung 2020. 2020, Bundesministerium für Soziales, Gesundheit, Pflege und Konsumentenschutz Geschäftsführung der Bundesgesundheitsagentur: Wien.

[102] Pilz M.J., Aaronson N.K., Arraras J.I. (2021). Evaluating the Thresholds for Clinical Importance of the EORTC QLQ-C15-PAL in Patients Receiving Palliative Treatment. J Palliat Med.

[103] Murtagh, F., Impact of the Integrated Palliative care Outcome Scale – Interpreting the Data. Presentation 18 April 2018. 2018, Hull York medical School.

[104] Zielsteuerung-Gesundheit on behalf of Bundesministerium für Soziales Gesundheit Pflege und Konsumentenschutz, FACT SHEET & METHODISCHE ÜBERSICHT Outcome-Messung: Hospiz- und Palliativversorgung (Palliativstationen). 2020, Zielsteuerung-Gesundheit: Wien.

[105] Deutsche Gesellschaft Fur Palliativmedizin, Nationales Hospiz-unf Palliativregister. Glossar 2019.

[106] Ministry of Health Labour and Welfare. List of Statistical Surveys conducted by Ministry of Health, Labour and Welfare. 2021 [Accessed: 7 April 2021]; Available from: https://www.mhlw.go.jp/toukei/itiran/eiyaku.html.

[107] Foundation for Promotion of Cancer Research. Cancer Statistics in Japan-2019. 2019 [Accessed: 7 April 2021]; Available from: https://ganjoho.jp/data/reg_stat/statistics/brochure/2019/cancer_statistics_2019.pdf.

[108] Cancer Information Service [ganjoho.jp]. Latest Cancer Statistics. 2021 [Accessed: 7 April 2021]; Available from: https://ganjoho.jp/reg_stat/statistics/stat/summary.html.

[109] Hospice NZ. The Year in Review 2019-2020. [Accessed: 20 December 2020]; Available from: https://www.hospice.org.nz/about-hospice-nz/the-year-in-review-2019-2020/.

[110] Axelsson L., Alvariza A., Lindberg J., Öhlén J., Håkanson C., Reimertz H. (2018). Unmet palliative care needs among patients with end-stage kidney disease: A national registry study about the last week of life. J Pain Symptom Manage.

[111] Martinsson L., Heedman P.A., Lundström S., Axelsson B. (2017). Improved data validity in the Swedish Register of Palliative Care. PLoS One.

[112] Eager K., Watters P., Currow D., Aoun S., Yates P. (2010). The Australian Palliative Care Outcomes Collaboration (PCOC) - measuring the quality and outcomes of palliative care on a routine basis. Aust Health Rev.

[113] Leemans K. (2015). Quality indicators for palliative care services: Mixed-method study testing for face validity, feasibility, discriminative power and usefulness. Palliat Med.

[114] Leemans K. (2013). Towards a standardized method of developing quality indicators for palliative care: protocol of the Quality indicators for Palliative Care (Q-PAC) study. BMC Palliat Care.

[115] Leemans K. (2017). Systematic Quality Monitoring For Specialized Palliative Care Services: Development of a Minimal Set of Quality Indicators for Palliative Care Study (QPAC). Am J Hosp Palliat Care.

[116] Danish Clinical Quality Program– National Clinical Registries (RKKP). RKKP: In English. [cited 2020 19 November 2020]; Available from: https://www.rkkp.dk/in-english/.

[117] Nationales Hospiz und Palliatiav Register. Welcome to the National Hospice and Palliative Care Register. 2020; Available from: https://www.hospiz-palliativ-register.de/.

[118] Svenska Palliativ Registret, Årsrapport för Svenska palliativregistret 2019. 2020.

[119] NHS Benchmarking Network, National Audit of Care at the End of Life: Second round of audit report - Northern Ireland. 2020.

[120] WA Cancer and Palliative Care Network for the WA Department of Health, WA End-of-Life and Palliative Care Strategy 2018–2028. 2018, WA Department of Health.

[121] Palliative Care Australia, Palliative Care Service Development Guidelines. 2018: Canberra.

[122] Canadian Partnership Against Cancer, Person-Centred Perspective Indicators in Canada: A Reference Report. Palliative and End-of-Life Care. 2017, Canadian Partnership Against Cancer: Toronto, Ontario.

[123] Canadian Partnership Against Cancer, Palliative and End-of-Life Care: A Cancer System Performance Report. Techincal Appendix. 2017, Canadian Partnership Against Cancer: Toronto, Ontario.

[124] Ministere des Solidarites et de la Sante. Launch of preparatory work for the development of the next National plan for the development of palliative care and support at the end of life. 2020 26 February 2021]; Available from: https://solidarites-sante.gouv.fr/actualites/presse/communiques-de-presse/article/lancement-des-travaux-preparatoires-a-l-elaboration-du-prochain-plan-national.

[125] Currow D.C., Eagar K., Aoun S., Fildes D., Yates P., Kristjanson L. (2008). Is It Feasible and Desirable to Collect Voluntarily Quality and Outcome Data Nationally in Palliative Oncology Care?. J Clin Oncol.

[126] Leitlinienprogramm Onkologie, S3-Guideline Palliative care for patients with incurable cancer Short version 1.0 2015.

[127] Nationales Hospiz und Palliatiav Register. Welcome to the National Hospice and Palliative Care Register. 2020 3 February 2021]; Available from: https://www.hospiz-palliativ-register.de/.

[128] Health Service Executive, Palliative Care. Key Performance Indicator Metadata 2019: Dublin, Ireland.

[129] Hull York Medical School. ACCESS RESOLVE TRAINING. 20 May 2021]; Available from: https://www.hyms.ac.uk/research/research-centres-and-groups/wolfson/resolve/access-resolve-training-resources.

[130] Blanchard M., Burns S., Allingham S., Clapham S., Daveson B., Eagar K. (2020).

[131] Cohen J. (2021). Nationwide evaluation of palliative care (Q-PAC study) provided by specialized palliative care teams using quality indicators : Large variations in quality of care. Palliat Med.

[132] Martinsson L., Fürst C.J., Lundström S., Nathanaelsson L., Axelsson B. (2012). Registration in a quality register: a method to improve end-of-life care–a cross-sectional study. BMJ Open.

[133] Etkind S.N. (2015). Capture, transfer, and feedback of patient-centered outcomes data in palliative care populations: does it make a difference? A systematic review. J Pain Symptom Manage.

[134] Murtagh, F., The UK experience of implementing Palliative Care Outcome Measures. Presentation. 2017, Wolfson Palliative Care Research Centre and Hull York Medical School: London, UK.

[135] Dudgeon D. (2018). The Impact of Measuring Patient-Reported Outcome Measures on Quality of and Access to Palliative Care. J Palliat Med.

[136] Gholiha A., Fransson G., Fürst C.J., Heedman P.A., Lundström S., Axelsson B. (2011). Stora luckor i journaler vid vård i livets slutskede: journaluppgifter saknas för data inrapporterade till Palliativregistret [Big gaps in documentation of end-of-life care: incomplete medical records reported to the Swedish Registry of Palliative Care]. Läkartidningen.

[137] Dupont C. (2021). Pilot Study to Develop and Test Palliative Care Quality Indicators for Nursing Homes. Int J Environ Res Public Health.

[138] Marie Curie, Palliative care and the UK nations. An updated assessment on need, policy and strategy. Implications for Wales. 2016, Marie Curie.

[139] Lindskog M., Tavelin B., Lundström S. (2015). Old age as risk indicator for poor end-of-life care quality – A population-based study of cancer deaths from the Swedish Register of Palliative Care. Eur J Cancer.

[140] van Esch T.E.M., Coppen R., Engels Y., Reyners A., van der Heide A., Francke A.L. (2018). Meer inzicht in de kwaliteit van palliatieve zorg door koppeling van routinematige registraties – is dat haalbaar?. Nederlands-Vlaams Tijdschrift voor Palliatieve Zorg.

[141] Piili, R., End-of-Life Decision-Making in Cancer Patients. Attitudes, ethics and background factors among Finnish physicians and medical students. 2019, Tampere University.

[142] Health Canada, Framework on Palliative Care in Canada. 2018, Health Canada: Ottawa, Ontario.

[143] Health Canada, Action Plan on Palliative Care. Building on the Framework on Palliative Care in Canada. 2019, Health Canada: Ottawa,Ontario.

[144] Palliative Care Australia, National Palliative Care Standards. 2018: Canberra.

[145] Public Health England (PHE) the National Council for Palliative Care (NCPC) and Hospice UK, Joint Statement on the Minimum Data Set for Specialist Palliative Care Services. 2017, The National Council for Palliative Care.

[146] Currow D.C., Allingham S., Yates P., Johnson C., Clark K., Eagar K. (2015). Improving national hospice/palliative care service symptom outcomes systematically through point-of-care data collection, structured feedback and benchmarking. Support Care Cancer.

[147] Sen M., McGwyne H. (2017). The implementation of OACC in a hospice setting. BMJ Support Palliat Care.

[148] Murtagh, F., Learning from the use of outcome measures: the difference we can make. Presentation. 2015, Cicely Saunders Institute Department of Palliative Care, Policy & Rehabilitation, King’s College London: London, UK.

[149] Leemans K. (2015). How to implement quality indicators successfully in palliative care services: perceptions of team members about facilitators of and barriers to implementation. Support Care Cancer.

[150] Department of Health. Quality and outcomes framework (QOF) statistics - annual report. 19 March 2021]; Available from: https://www.health-ni.gov.uk/articles/quality-and-outcomes-framework-qof-statistics-annual-report.

[151] Foreit K, Moreland S, LaFond A. Data Demand and Information Use in the Health Sector, Conceptual Framework [MS-06-16a]. Chapel Hill, NC: MEASURE Evaluation; 2006; Available from: https://www.measureevaluation.org/resources/publications/ms-06-16a.html.

