# Supplementary material for: What gets measured in palliative care? A review and synthesis of routine data collection in 16 countries
Source: Health Policy Open. 2025 Apr 19;8:100141. doi: 10.1016/j.hpopen.2025.100141 (PMC12083991; doi:10.1016/j.hpopen.2025.100141)
Supplement: Supplementary Data 1 [file mmc1.docx]

# Additional File 1. Overview of the palliative care data infrastructure in the countries reviewed

| Country | Overview of current palliative care data infrastructure |
| --- | --- |
| Australia | (1) The Australian Institute of Health and Welfare governs 'Palliative care services in Australia', an online web report on the activity and characteristics of palliative care services across Australia, which is updated twice a year.  (2) Palliative Care Outcomes Collaborative (PCOC) - is a national longitudinal database, populated by SPC services. Benchmarking - with nationally agreed upon quality and outcomes measures - is at a national, jurisdictional or peer group level. Benchmarks are presented separately for services in hospital/hospice and community setting. The collaborative represents approx. 85% of all palliative care patients referred to specialist services. |
| Austria | (1) Hospice Austria collates detailed information annually on service delivered in hospices and palliative care facilities. The Austrian National Public Health Institute (Gesundheit Österreich GmbH (GÖG)) uses the national dataset to create yearly monitoring reports about the progress of hospice and palliative care provision.  (2) There has been a recent focus on embedding PROMs - initially in palliative care units. PROMs for pain and other symptoms have recently been added to the National Outcomes Measurement project, though they are yet to be reported upon given limited data sources. |
| Belgium | The End-of-Life Care Research Group at VUB-UGent has developed and implemented 'Quality indicators for palliative care (QPAC)' in Flanders - a set of 31 quality indicators on palliative care for specialised palliative care service, measured every six months using ongoing survey data. Palliative care teams use QPAC to measure and improve the quality of care in various aspects. This indicator set has recently been modified for use in nursing homes.  Researchers in Belgium make significant use of linking existing national administrative datasets to study and evaluate end-of-life care, including the development of population-level quality indicators for appropriateness of end-of-life care in cancer, COPD and dementia.  The Belgian Health Care Knowledge Centre (KCE), reports annually on the performance of the health system against 100 indicator, include four on end-of-life care. However, data are limited to terminal cancer patients, as nationwide data on end-of-life care is only available from the Cancer Registry.  The governments of the different regions of Belgium have mandated the nationwide implementation of the interRAI suite of instruments (e.g. the interRAI Home Care instrument, the interRAI Palliative Care instrument). The federal government in Belgian has developed online software in order to complete the instruments. This software (BelRAI 2.0) enables a transfer of client data between care professionals and health care settings.  A substantial project (2008) to derive a web-based Minimum Dataset for palliative care to be used across all settings was not continued. |
| Canada | The Canadian Institute for Health Information (CIHI) provides comprehensive data on access to palliative care using existing data from: acute care, complex continuing care, emergency departments, long-term care facilities, home care, physician billings, publicly funded drug programs and human resources.  Health Quality Ontario and the Ontario Palliative Care Network, as well as research groups, make substantial use of linking existing national administrative datasets, including those mentioned above.  The Canadian Partnership against Cancer is leading efforts to collect more pan-Canadian data on palliative care and end-of-life care. It funded a three-year project to develop a common set of indicators for PROMs and PREMs during cancer treatment, including palliative care. |
| Denmark | The Danish Palliative Care Database (DPD) (Dansk Palliativ Database) is a national quality of care clinical database, approved by the Danish Board of Health. It is mandatory for all specialty palliative care units to register all referred patients in the DPD (including those referred to and not admitted). |
| Finland | Makes significant use of existing national registries as well as a Minimum Dataset in long-term care (LTC) created using the Resident Assessment Instrument (RAI), which facilitates examination of 26 performance measures to monitor quality of care in LTC (not specific to palliative care). It will become mandatory for LTCs to participate in the RAI from 2023. |
| France | A recently established National Centre for Palliative Care (Centre national des soins palliatifs et de la fin de vie) collects, analyses and publishes data on end-of-life support and palliative care at the national and regional level. It makes use of the French national health system database (Système National des Données de Santé, SNDS), along with other sources of data to provide information on access to, and quality of palliative and end-of-life care in France. |
| Germany | The HOPE/National Hospice and Palliative Care Register (DGP Nationales Hospiz- und Palliativregister) is a cross-sectional, multi-centre and nationwide patient register, populated using data collected during an annual sample period of 3 months or 30 patients. Palliative and hospice care institutions, such as inpatient palliative care units, hospices, or specialised outpatient palliative care teams participate; though it is not mandatory. |
| Ireland | The SPC Minimum Dataset (MDS) is a national survey of demographic and patient activity data for SPC services. Metrics are submitted monthly by four SPC services: inpatient units, community (homecare) services, day care services and acute hospitals. Participation by the services is mandatory. |
| Japan | Makes extensive use of existing administrative datasets (primarily by researcher groups), and cross-sectional surveys conducted by the Ministry of Health, Labour and Welfare (MHLW), the National Cancer Center Japan, the Japanese Society for Palliative Medicine, and research organisations. |
| New Zealand | Hospice NZ undertakes an annual survey of its members covering: service data, financial data, and community services support data (education and clinical advice and support). The organisation is currently in the process of developing a 'Hospice Data Commons', which will see services uploading data on a quarterly basis. Analysis will focus on descriptive indicators, trends, indirect support (i.e. hospice reach in the community) and 'standards' indicators.  While InterRAI palliative care assessments in aged residential care have been mandatory since 2015, there is a lack of published information available on the current utilisation of these assessments.  A recent substantive research project titled 'Trajectories of Care at the End of life in New Zealand’ made use of NZ's unique health identifier to link numerous administrative data sources to provide intelligence on end-of-life care and service utilisation. |
| Norway | While there is no register specific to palliative care in Norway, the Cancer Registry has several clinical registries; several of which have started or are starting to include quality of life measures and patient reported outcome measure (PROMS), e.g., the Breast Cancer Registry and the Colorectal Cancer Registry.  The SPC Services, which serve as training, centres for physicians seeking competence training in palliative care, complete detailed surveys each year for the Directorate of Health.  Researchers in Norway led the development the EAPC basic dataset for reporting patient characteristics and medical variables in palliative care cancer research. |
| Sweden | The Swedish Register of Palliative Care (SRPC) (Svenska palliativregistret), is a national quality register, which holds information on the quality of care prior to death, regardless of where care is given, or where death occurs. It collects data from hospitals, hospices, nursing homes and home care, with an end of life questionnaire (ELQ) about palliative care, in the last week of life, independent of diagnosis.  Registration occurs after death, and the register contains information on approximately 60% of all deaths each year. |
| Switzerland | SwissPALL 3.0 is currently being undertaken to provide a longitudinal palliative care cohort via a web-based platform (modelled on PCOC), to be populated by all certified SPC institutions. |
| The Netherlands | The Palliative Care Information System (Informatiesysteem Palliatieve Zorg), which was recently developed, makes use of existing linked data from national datasets. The system currently involves linked data from three data sources: from Statistics Netherlands about causes of death and background characteristics of people with a death cause relevant for palliative care, Nivel Primary Care Database and Dutch Hospital Data. There is potential to include further data sources in further iterations of the system. |
| United Kingdom | The UK does not have a national dataset for palliative care. The Outcome Assessment and Complexity Collaborative (OACC) project, and more recently the RESOLVE programme, introduces outcome measures into palliative care services to measure, demonstrate and improve care for patients and their families.  The National Minimum Data Set collection for SPC Services (MDS) which was developed in 1995, was discontinued in 2017, citing the lack of funding required to significantly update and enhance the dataset. |
| England | Palliative and End-of-life care Profiles have been developed by Public Health England, to improve the availability and accessibility of information and intelligence around palliative and end-of-life care. Public Health England also produces an 'Atlas of variation for palliative and end-of-life care in England', to highlight geographical variation in access to services.  England participates in the National Audit of Care at the End of life (NACEL), which is a national, comparative, annual audit of the quality and outcomes of care experienced by the dying person and those important to them during the last admission leading to death in acute, community hospitals and mental health inpatient facilities, in England, Wales and Northern Ireland.  General Practitioner (GP) practices are incentivised through their General Medical Services (GMS) contract Quality and Outcomes Framework (QOF) to establish and maintain a Palliative Care Register of all patients in need of palliative care/support.  A Palliative Care Clinical Data Set (PCCDS), to provide patient-level palliative care data was developed and piloted in 2015, but not implemented nationally owing to a lack of funding, |
| Northern Ireland | Northern Ireland participated in the UK's National Minimum Data Set collection for SPC Services (MDS) until its discontinuation in 2017  NI participates in the National Audit of Care at the End of life (NACEL) (see above).  GP practices in NI are also incentivised to establish and maintain a Palliative Care Register as part of their Quality and Outcomes Framework (QOF). |
| Scotland | There has been a recent focus on harnessing existing individual-level administrative datasets to provide the intelligence required for planning palliative care services. Public Health Scotland publishes official statistics on a suite of Integration Indicators which all the Health and Social Care Partnerships are required to report on annually. Specific to palliative care is the indicator ' Percentage of End of life Spent at Home or in a Community Setting'.  Scotland has a primary care Palliative Care Register. GPs are incentivised under the 'Primary Medical Services Directed Enhanced Services (Scotland) 2019 Palliative Care Scheme', to identify and record patients who may benefit from palliative and end-of-life care, and to adopt a palliative care approach to their care. Clinical data of those who consent to being in the register can be shared with relevant parties. |
| Wales | Wales participated in the UK's National Minimum Data Set collection for SPC Services (MDS) until its discontinuation in 2017.  Wales has seen significant recent developments in its health data infrastructure, with the introduction of the Welsh Clinical Portal. Information on SPC assessment for cancer patients (as well as non-cancer patients once referred to specialist services), which was previously held in Cancer Network Information System Cymru (CaNISC), will shortly migrate to this portal.  As with the other UK nations, GPs are incentivised - as a part of a Quality Assurance and Improvement Framework - to establish and maintain a Palliative Care Register, which identifies and records people with palliative care needs, and assists in the delivery of multi-disciplinary palliative care.  Wales participates in the National Audit of Care at the End of life (NACEL), described above. |
