# Supplementary material for: What gets measured in palliative care? A review and synthesis of routine data collection in 16 countries
Source: Health Policy Open. 2025 Apr 19;8:100141. doi: 10.1016/j.hpopen.2025.100141 (PMC12083991; doi:10.1016/j.hpopen.2025.100141)
Supplement: Supplementary Data 2 [file mmc2.docx]

# Additional File 2. Considerations to improve the palliative care data infrastructure in the countries reviewed

| Country | Details |
| --- | --- |
| Australia | In Australia, until now it has not been feasible to link PCOC with other healthcare data. This issue is being resolved in the next iteration of data collection (version 4), where it has been agreed in principal, that there will be identifiers which will facilitate linkage to the rest of the health care system, via probabilistic linkage [1].  In NSW, efforts are under way to implement the use of a 'unique identifier' for everyone in the State, which would render it much more feasible to link across the health system, with appropriate ethical approval [1]. PCOC has a place holder for the use of such a unique health identifier.  The collaboration also identifies areas for future research: understanding why similarly resourced services have different patient outcomes; understanding why pain is the only symptom not to significantly improve, examining of whether service level improvements are translating into improvements at an individual patient level (at a level that is clinically meaningful) [2]. |
| Austria | (1) In Austria, Hospice Austria aims to incorporate basic palliative care in primary care services such as acute hospitals, long term care facilities, home nursing/home care and GP’s, and considers to start to provide data on these endeavors.  (2) It is expected that as soon as PROMs are sufficiently established in Austria, the indicators that can be collected based on PROMs (PROMs for pain, PROMs for shortness of breath) will be reported upon [3]. It is expected that in the future, considerations will be given to extending the outcome measurement to palliative care teams and inpatient hospices, not just palliative care wards. |
| Belgium | In Belgium, the QPAC indicators has been approved by the Flemish Institute for Quality of Care for regional quality monitoring and public reporting on palliative care. QPAC has recently been expanded to and adapted for nursing homes, and these quality indicators will be added to the regional dataset for palliative care by the Flemish Institute for quality of care in 2021.  There is a recommendation that an e-application should be developed to enable the service to enter data themselves and to immediately request the results of their quality measurements via a personal dashboard with their own quality results and benchmark data. Ideally, this e-application will be linked to existing electronic patient records[4]. For the palliative home care teams, a separate module to measure the quality indicators is already implemented into their electronic patient files.  Effort continue to refine the method used to risk adjustment, to correctly compare teams, and ensure the quality results are transparent to the sector. which risk factors are best measured or registered so that a more correct comparison is possible [4]. |
| Canada | Health Canada's framework for palliative care in 2018 [5], and its associated action plan [6], highlighted research and the collection of data on palliative care as one of four priority areas for action, and set out goals for this priority area: (1) develop and promote the use of standardised person- and family-reported outcomes and experience measures, as well as screening and assessment tools across all settings. (2) Develop precise indicators related to palliative care, including distinctions based approaches. (3)Work with national/international survey developers to add palliative care questions, including information on community care. (4) Use existing big data sources for more than one purpose and link databases to improve efficiencies in data collection and analysis. (5) Improve data collection on the uptake and consistency of advance care plans, focusing on where, how and by whom they are most used.  The Canadian Partnership against Cancer is leading efforts to collect more pan-Canadian data on palliative care and end of life care, and has funded a three year project to develop a set of indicators for PROMs and PREMs during cancer treatment, including palliative care. |
| Denmark | The findings of a research project, where the changes over time in patients' symptoms and problems were analyzed will lead to the development of new indicators based on changes in patients' symptoms and problems over time. Work has been undertaken to provide evidence to assess what variables and indicators will need to be added to enable assessment of changes in patients symptoms and problems over time[1]. |
| Finland | In Finland, the Ministry of Social Affairs and Health have named a project group to plan a national palliative care database, co-ordinated by. THL is coordinating the project, which is in very early the stages of planning [1]. The Ministry has also commissioned a further project on the status of palliative care and convalescent care in Finland, and the development of quality indicators for palliative care is part of this project.  Currently, participation in interRAI is not mandatory. This will change from 2023, when all municipalities must start using RAI assessment. |
| Ireland | In terms of palliative care outcomes, a comprehensive economic evaluation of palliative care in Ireland, called for specialist palliative care services to promote and participate in the development of quality indicators and patient reported outcome measures of services on a national basis [7]. PCOC is currently being tested in a selection of specialist palliative care service. Other aspects have been highlighted as requiring action:   - Review the implementation of the current MDS, including clarifying of definitions, capturing referrals not seen, review the use and value of current data collected and consider if this should be refined including reduced, as much of the data is not used. - Capitalize on the use of existing administrative data and improve data capture at this level   - Consider use of diagnostic codes, such as ICD 10, with attendant training needs of voluntary palliative care service providers   - Consider use of Diagnostic Related Groups   - Consider use of other markers of complexity, such as Charlson Co-morbidity Index - Develop the Unique Health Identifier to allow more accurate recording of specialist palliative care service use |
| Japan | In Japan, data on end-of-life care and palliative care and academic studies based on such data are accumulated at the national and private levels, but only partially and not yet comprehensively. Comprehensive data on member institutions of the Hospice Palliative Care Japan have been developed and published annually. Still, some non-member institutions and institutions that do not publish data have been pointed out. Furthermore, although experts have pointed out the importance of paediatric palliative care and hospice, there is no national strategy and no comprehensive data collection. Experts have called for central government to develop comprehensive data, including in paediatric palliative care and hospice. |
| New Zealand | Hospice NZ are in the process of developing a 'Hospice Data Commons' that will deliver insight and value for hospices across New Zealand. All hospices will collect and contribute common data based on agreed criteria on a regular basis. The information will be stored in a secure common database with appropriate security and privacy provisions. It will be regularly analysed using automated systems and information presented back to hospices in a dashboard format. Standards indicators – that relate to the Hospice NZ Standards for Palliative Care 2019, will be applied to the data. Hospice NZ will establish ongoing dedicated data governance and oversight for the common data infrastructure. As at March 2021, the Hospice Data Commons has reached the milestone of data upload testing. The aim is to have the first full data upload by hospices commencing towards the end of 2021 [1]. |
| Norway | The last national expert report on palliative care recommended to establish a national quality register for palliative care, but the government did not support this recommendation. However, three national projects are being undertaken that will also impact on palliative care: 1. ‘Akson electronic medical record’ – one common electronic patient record across all levels and institutions. 2. The Norwegian Health Data Program – a project to harmonize all existing registers to facilitate reporting, quality assurance, research and development. The project includes the establishment of 3. A National Health Analysis Platform, to be launched in the spring of 2022. |
| Sweden | In Sweden, there are plans to improve data capture by transferring data directly from the medical records into the register, which would improve the validity of the data entered in the SRPC, especially as pertains to symptom prevalence. This would also reduce the administrative burden involved in populating the dataset. However, it is recognised that the quality of the data capture will be dependent on the quality of the information in the patients' medical records. In the autumn of 2019, the Swedish Palliative Care Register's steering group began planning for an update of the related party survey [8]. This updated version is now in operation with a new output portal on the home page dedicated to this [1]. |
| Switzerland | In Switzerland, the establishment of a database (Swiss PALL) comparable to other national specialist palliative care datasets and following national quality indicators has become one of the priorities of the Swiss Society for Palliative Care [1]. A new version of a MDS, SwissPALL (version 3.0) with its consolidated dataset is under review, and a data host for future collaboration offering secured data will be selected shortly [1]. |
| The Netherlands | In the Netherlands, it is envisaged that the information system which currently includes data from three administrative datasets will be expanded to include more data sources. Technical advances are being considered which could increase the willingness of healthcare organisations to provide data via the use of a ‘personal health train construction'. In this scenario, rather than bringing the data together in a central database (as is currently the case in the project phase), the algorithms of the analysis (as trains) are transferred via a technical infrastructure (the rails) to the various data sources (the stations). Because the data then remains with the owner of the data, this could possibly increase the willingness to cooperate on the part of healthcare organisations that have to provide data [9]. |
| United Kingdom | The OACC project team are closely involved in the development of case-mix classification, so that, in the longer term, case-mix adjustment of outcome measurement becomes possible, and this will enable realistic benchmarking of similar services useful to services and commissioners alike [10]. Benchmarks have recently been established in the RESOLVE project. RESOLVE is working to establish a Palliative Care Outcomes Registry in the UK. Hospice UK continues to support this work with regular ECHO webinars, and workshops. |
| Northern Ireland | In Northern Ireland the Palliative Care in Partnership Programme is working within the Health and Social Care system on the regional roll-out of a new integrated digital healthcare record for patients. This initiative, ENCOMPASS, is commencing development in September 2021 with the palliative care work-stream as an early adopter. This new integrated digital information initiative will provide a more comprehensive palliative care information dataset within the hospital and community services and for those patients receiving care in the independent hospice sector [1].  Similarly, work has commenced on developing access to data in primary care under the GPIP (General Practice Information Platform) initiative. This should support improved identification of patients with palliative care needs in the community through enhanced access to information on primary care clinical systems [1]. |
| Scotland | The Scottish Partnership for Palliative Care published a report *‘Every Story’s Ending – proposals to improve people’s experiences of living with serious illness, dying and bereavement in Scotland’*, in 2021, in consultation with stakeholders [11]. A chapter in the report explores how to improve the measurement of outcomes and quality of experience of dying, in Scotland. Triangulation of data sources is vital, including complementing routinely collected data (e.g., Key Information Summaries) which are readily available at the local level, with patient and carer surveys. The report sets out several recommendations in relation to next steps for palliative care data infrastructure in Scotland: the establishment of a Palliative Care Outcomes Group; the development of a national survey of bereaved informal care givers; the expansion of the role of digital products; the development of local data and measures and support to Health and Social Care Professionals to understand the mortality landscape for their population, using both quantitative and qualitative measures. |
| Wales | There is a project underway to identify core outcome domains which will underpin a dataset for assessment of service quality, along the lines of England’s OACC and Australia’s PCOC [1] which was issued as a recommendation in the 2016 Marie Curie report [12]. The report recommended development of outcome measure reporting structured in a similar way to that operated in Australia (PCOC) and building on the work being led by the Cicely Saunders Institute (Outcome Assessment and Complexity Collaborative – OACC and RESOLVE [12].  The electronic Palliative Care Casenote, which logs multidisciplinary meeting outputs, assessments and communications, is due to transition from a stand-alone oncology electronic record system to an all Wales Clinical Portal, and will allow greater access to healthcare professionals across all care settings. It will integrate within the National Data Record for Wales which will facilitate data sharing and capture of outcomes [1]. |

**References**

1. Personal Communication with Expert Reviewer. 2021.

2. Currow, D.C., Allingham, S., Yates, P., Johnson, C., Clark, K., Eagar, K., *Improving national hospice/palliative care service symptom outcomes systematically through point-of-care data collection, structured feedback and benchmarking.* Support Care Cancer, 2015. **23**(2): p. 307-15.

3. Zielsteuerung-Gesundheit on behalf of Bundesministerium für Soziales Gesundheit Pflege und Konsumentenschutz, *Outcome-Messung im Gesundheitswesen basierend auf dem Mess- und Vergleichskonzept. Detailanalyse relevanter Outcomes im Gesundheitswesen. Aktualisierte Fassung 2020*. 2020, Bundesministerium für Soziales, Gesundheit, Pflege und Konsumentenschutz Geschäftsführung der Bundesgesundheitsagentur: Wien.

4. End-of-Life Care Research Group, *Kwaliteitsindicatoren voor palliatieve zorg. Waar zijn we en wat is de toekomst? Beleidsrapport [Quality indicators for palliative care. Where are we and what is the future? Policy report]*. 2017, End-of Life Care Research Group (VUB and UGent): Flanders, Belgium.

5. Health Canada, *Framework on Palliative Care in Canada*. 2018, Health Canada: Ottawa, Ontario.

6. Health Canada, *Action Plan on Palliative Care. Building on the Framework on Palliative Care in Canada.* 2019, Health Canada: Ottawa,Ontario.

7. Brick, A., O'Hara, S., Normand, C., Smith,S., et al, *Economic Evaluation of Palliative Care in Ireland Final Report*. 2015, Trinity College Dublin: Dublin

8. Svenska Palliativ Registret, *Årsrapport för Svenska palliativregistret 2019*. 2020.

9. Francke, A., Oosterveld-Veld, M., Boddaert, M., Engels, Y .,van der Heide, A., Heins, M., Onwuteaka-Philipsen, B., Verheij, R., Reyners, A., *Leren van bestaande data over palliatieve zorg. Kwaliteitsindicatoren, zorggebruik en een governance structuur voor het Informatiesysteem Palliatieve Zorg*. 2020, NIVEL, ZonMw and PalZon: Utrecht, The Netherlands.

10. Witt, J., de Wolf-Linder, S., Dawkins, M., Daveson, BA., Higginson, IJ., Murtagh, FEM. , on behalf of the Outcome Assessment and Complexity Collaborative (OACC) Team., *Introducing the Outcome Assessment and Complexity Collaborative (OACC) Suite of Measures A Brief Introduction - Version 2*. 2015, Department of Palliative Care, Policy and Rehabilitation,Cicely Saunders Institute, King’s College London.

11. Scottish Partnership for Palliative Care, *EVERY STORY’S ENDING. Proposals to improve people’s experiences of living with serious illness, dying and bereavement in Scotland.* 2021: Edinburgh, Scotland.

12. Marie Curie, *Palliative care and the UK nations. An updated assessment on need, policy and strategy. Implications for Wales*. 2016, Marie Curie.
