# Supplementary material for: What gets measured in palliative care? A review and synthesis of routine data collection in 16 countries
Source: Health Policy Open. 2025 Apr 19;8:100141. doi: 10.1016/j.hpopen.2025.100141 (PMC12083991; doi:10.1016/j.hpopen.2025.100141)
Supplement: Supplementary Data 3 [file mmc3.docx]

Supplementary Table 1 Patient level databases specific to palliative care

| Country  (database) | Description |
| --- | --- |
| Australia  (Palliative Care Outcomes Collaboration - PCOC)  [11,12] | PCOC is a national palliative care outcomes and benchmarking programme. A core part of the programme is to create a national longitudinal database for palliative care which captures information across a patient’s disease trajectory to facilitate improved patient outcomes. |
| Denmark  (Danish Palliative Care Database – DPD)  [13,14] | DPD is a national quality of care clinical database. It is mandatory for all Specialty Palliative Care (SPC) units in Denmark to register all referred patients in the DPD, which they do within three days of the patient being admitted to the SPC. The register also includes data on patients referred to but not admitted to the service. |
| Germany  (Nationales Hospiz- und Palliativregister - HOPE)  [15-18] | A cross-sectional, multi-centre and nationwide register. It collates patient-related data from the day-to-day provision of care in hospice and palliative care settings across regions, professions and sectors at least once a year for a minimum random sampling period of three months and a sample size of 30 patients. HOPE is a modular system with a core database and a variety of optional modules. It includes a basic core database as well as additionally modules, assessed by medical staff at the beginning of the care segment of hospice and palliative care and at the end of hospice and palliative care or of the respective care segment/uninterrupted care period. |
| Sweden  (The Swedish Register of Palliative Care - SRPC)  [19-21] | The SRPC is a national quality register which holds information on the quality of care prior to death, regardless of where care is given, or where death occurs. It collects data from hospitals, hospices, nursing homes and home care, focusing on the last week of life, independent of diagnosis. Registration occurs after death, and the register contains information on approximately 60% of all deaths each year. |
| Switzerland  (SwissPALL)  [22-24] | A project has been underway for quite some time to develop a national patient database using a web-based platform (modelled on PCOC) which would be owned by institutions/participants. SwissPALL is currently in its third iteration and aims to include all certified SPC institutions including mobile teams and specialist long-term care facilities. A consolidated database is under review, and a data host for future collaboration offering secured data will shortly be selected. |
| United Kingdom  (The Outcomes and Complexity Collaboration – OACC and the RESOLVE programme)  [25-26] | These consist of a suite of patient reported outcome measures. The purpose of the measures are to improve day to day clinical care; enable better informed strategic, management, service decisions locally, sector wide, nationally; serve more than one purpose, i.e. capture complexity, enable individual care to be improved, but also deliver quality improvement for services, and enable national outcomes, funding and a database. The RESOLVE programme is working to establish a Palliative Care Outcomes Registry for the United Kingdom. |
| United Kingdom  (General Practice Care Registers)  [27-30] | In each of the four United Kingdom nations, General Practice (GP) are incentivised under their individual Quality Outcome Frameworks to establish and maintain palliative care registers, identifying and recording patients who may benefit from palliative and end of life care. These GP registers cover all patients with a life limiting condition, identified as benefitting from a palliative approach to their care. However, these GP registers are not collated nationally. |

Supplementary Table 2 Palliative care services activity databases

| Country  (database) | Description |
| --- | --- |
| Austria  (Database of Hospice Austria)  [40] | Hospice Austria has been collating information annually since 2005, from hospices and palliative care on demographic information of the patient, the structure of staff at individual services, activities of services, and other data on structures of service delivery. |
| Ireland  (Minimum Data Set - MDS)  [41-42] | The Health Service Executive’s Minimum Data Set (MDS) is a national survey of demographic and patient activity data for SPC services. All services at the in-patient, community and day care level are required to return monthly data. In-patient SPC refers to care received by those admitted to specialist palliative care (SPC) services as an in-patient in a hospice or in an acute hospital that provides specialist palliative care beds. Community SPC is provided to people in their normal place of residence by members of an SPC team and is also known as ‘homecare’. While day care SPC is defined as a short-term (day) admission to a service that provides care and support to patients with a serious illness. |
| New Zealand  (Database of Hospice NZ)  [43] | Hospice New Zealand’s annual service data cycle provides an understanding about hospice services, from the perspective of activity. The hospices submit data for analysis for both direct patient data, and community services data, covering service data, financial data, and community services support data (education and clinical advice and support). |

**Supplementary Table 3 Secondary use of existing administrative data**

| Country | Description |
| --- | --- |
| Belgium  [44-46] | Researchers in Belgium have worked extensively on linked existing administrative databases to study access to, and evaluate the quality of palliative and end-of-life care, including the development of population-level quality indicators for appropriateness of end-of-life care in cancer, COPD and dementia. Data are obtained from eight routinely collected population-level databases, consisting of: (1) the socio-demographic database of all individuals with healthcare insurance (2) the health care database containing all reimbursed health care use data on home, nursing home, outpatient and hospital care; (3) the pharmaceutical database containing all reimbursed medication data; (4) Belgian Cancer Registry data with diagnostic information on all incidences of cancer (5) death certificate data containing cause of death; (6) population registry data including nationality and household composition; (7) census data, and (8) the fiscal database. |
| Canada  [47] | In Canada, the Canadian Institute for Health Information makes significant used of administrative databases to provide a comprehensive account of: access to palliative care location of death (in hospital/at home/in other health facility); access to palliative care in long-term care facilities; potentially preventable hospital care in the last month of life palliative care; location of death for people who were home care clients in their last year of life; and proportion of people who received palliative care in the last year of life, by diagnosis and setting. Data sources include acute care, complex continuing care, ED, long-term care facilities, home care, physician billings, publicly funded drug programs and human resource. |
| France  [48-50] | In France, the National Centre for Palliative care (CNSPFV), was set up in 2016, and is responsible for collecting data on palliative care and participating in the monitoring of public policies. The CNSPFV published its first edition of the National Atlas of Palliative Care and End of Life in 2018, and the second in 2020. The atlas uses a multitude of data sources including recurrent national surveys as well as administrative data, which is collected, analyzed and consolidated by different institutions. It presents demographic and health data based on renewed indicators, which makes it possible to visualize the development and transformation of the health care service and the needs of patients in relation to this service. The Atlas reports on indicators originally derived for the 2018 report as well as additional indicators. Examples include: number of palliative care beds (and beds in palliative care units) per 100,000 inhabitants (per region); places of death in 2018; distribution of patients who died in 2016 according to the modeling of end-of-life trajectories. |
| Japan  [51-54] | Researchers use the Japan National Administrative Healthcare Database, which contains linked data from different data sources: Vital Statistics, Survey of Medical Institutions, and Survey of Institutions and Establishments for Long-term Care, by municipalities, to study end-of-life care. For example, this database was used in a study to develop a methodology for measuring quality indicators of end-of-life care for cancer patients, and to assess the association between different types of long-term care services that home-dwelling older individuals utilized at their end of life, and place of death. The Japanese Diagnosis Procedure Combination (DPC) database is also used in palliative care research to examine the aggressiveness of end of life care (chemotherapy, intensive care unit admission and cardiopulmonary resuscitation) and procedures and prescriptions administered to cancer patients in the last 14 and 30 days of life. |
| New Zealand  [55-56] | The Ministry of Health commissioned a project to use national data linked by National Health Identifier number to identify the size of the primary palliative care work‐load, to be met by Primary Health Organisations and district nursing services. |
| The Netherlands  [57-59] | The new Palliative Care Information System (Informatiesysteem Palliatieve Zorg) makes significant use of linked data from existing national databases to provide a comprehensive description of specific domains of the quality of palliative care. The system currently involves linked data from three data sources: from Statistics Netherlands about causes of death and background characteristics of people with a death cause relevant for palliative care, Nivel Primary Care Database and Dutch Hospital Data. There is potential to include further data sources in further iterations of the system. |
| UK – England  [60-63] | Palliative and End-of-life Care Profiles have been developed by the National End-of-life care Intelligence Network, using a wide array of administrative data sources, to improve the availability and accessibility of information and intelligence around palliative and end-of-life care.The information is grouped into domains: place of death; underlying cause of death; mortality; death in usual place of residence; care homes and community; and hospital care. Along with interactive profiles showing palliative care statistics, data analysis reports are also published. Recent reports published in March 2020 include detailed reports of: 'Emergency admissions in the 3 months before death’ and '[Older people’s hospital admissions in the last year of life](https://www.gov.uk/government/publications/older-peoples-hospital-admissions-in-the-last-year-of-life)'. Additionally, 'The Atlas of variation for palliative and end-of-life care' is prepared in partnership with a wide range of organisations who provide access to existing data sources, to highlight geographical variation and to try to differentiate between warranted and unwarranted variation. |
| UK – Scotland  [64-66] | The recently established Integration Authorities routinely use data to inform their strategic commissioning activity, and have developed a good awareness and understanding of their local data. A newly developed system called 'Source', brings routinely-gathered information on health and social care to provide 'person-linked data' to assist with planning and commissioning. Source currently integrates hospital data from across NHS Scotland and the Health and Social Care Partnerships . Additionally, Public Health Scotland Official Statistics Publication on the quality outcome measure 'the percentage of last six months of life spent at home or in a community setting', by linking death data from the National Records of Scotland death records to hospital data. |
| UK – Wales  [67] | A number of existing data sources have been used to report on progress towards palliative care policy. Data sources include: the Cancer Informatics System; NHS Wales Informatics Service; Office for National Statistics; and the Patient Episode Data Wales. The information that can be obtained using these existing data sources includes: percentage of patients supported by a specialist palliative care (SPC) team; the number of deaths within 3 days of an emergency admission; percentage of deaths receiving SPC input by age group; percentage of deaths by place of occurrence (home; care home; hospice; hospitals; other); numbers of multiple emergency admissions for patients who have had contact with a SPC team; and average number of admissions, in the 12 months prior to death by cause of death. These metrics are not currently reported upon in a regular manner. |

Supplementary Table 4 Other sources of palliative care data

| Country | Description |
| --- | --- |
| **National surveys** |  |
| Finland  [68-70] | The Finnish Institute for Health and Welfare regularly undertakes surveys to provide a comprehensive account of the delivery of health and social care services. For example, the Elderly Services Status survey, which is undertaken every three years, examines round-the-clock care (enhanced service housing, service homes, nursing homes and wards in health centers) as well as home care. |
| France  [48,71] | The National Centre for Palliative care (CNSPFV) undertakes quantitative and qualitative surveys directly with USPs (hospital services made up of beds dedicated to palliative care and end-of-life support), and EMSP (multi-professional teams that support the care of patients at the end of their life, in hospitals, in medico-social establishments or at home) to collect information on the structures and human resources in palliative care. A comprehensive report of the most survey (October 2019) is available on the CNSPFV website (in French). This updated data has enabled CNSPFV to create an [interactive web map of palliative care resources](https://www.parlons-fin-de-vie.fr/carte-interactive-des-structures-de-soins-palliatifs/). |
| Japan  [72-74] | The Japan Hospice and Palliative care Evaluation (J-HOPE) studies are a series of large cross-sectional, anonymous, self-reported, questionnaire surveys among bereaved family members, which evaluate the 'quality of care' and 'quality of life' at Palliative Care Units by utilising the Care Evaluation Scale and Good Death Inventory, respectively. Furthermore, the annual survey which populates the Japanese Society for Palliative Medicine registry, includes data on structures, processes and outcomes from over 500 palliative care teams in Japan. |
| Switzerland  [75] | In 2018 the Office for Labour and Social Policy Studies carried out a survey on the status of palliative care in the cantons. The finding of the survey of the 26 cantons and 14 regions were presented in a 2019 report which detailed variation across cantons in the availability of specialist palliative care services in hospital care, long-term care and the outpatient sector [70]. |
| **Quality indicator datasets** | |
| Belgium  [76] | The Quality Register (QPAC) consists of a core set ("minimum indicator set") of 31 indicators that evaluate processes and outcomes of care in SPC services within the following eight domains of care: physical aspect of care; psychosocial aspects of care; information, communication and care planning with the patient; information, communication and care planning with family information, communication and care planning between care providers; type of care; and circumstances surrounding death coordination and continuity of care; and care for family. |
| **National audit** |  |
| United Kingdom England, Wales, Northern Ireland  [77-78] | National Audit for Care at the End of Life is a national, comparative, annual audit of the quality and outcomes of care experienced by the dying person and those important to them during the last admission leading to death in acute, community hospitals and mental health inpatient facilities, in England, Wales and Northern Ireland. It highlights how hospital care measures up to nationally agreed quality standards. All National Health Service acute, community hospital and mental health inpatient providers are eligible to register for the annual audit. |
| **Comprehensive clinical assessment tools (interRAI assessments)** | |
| Belgium  [22] | The governments of the different regions have mandated the nationwide implementation of the interRAI suite of instruments, and the federal government has developed online software, which enables a transfer of client data between care professionals and health care settings. |
| Finland  [69, 79] | The interRAI assessment (not the interRAI palliative care assessment module), is used to create a minimum database known as the THL RAI database, which facilitates a benchmarking project to assess the quality of long-term care. Data from the THL RAI database has been combined registry data to measure where elderly people died and what services they used before they die. Participation in interRAI assessments is not mandatory, though this was due to change in 2023, when all municipalities were due to start using RAI assessment. |
| New Zealand  [55,80] | In New Zealand, the interRAI assessments became mandatory in aged residential care from 1 July 2015 [50]. An InterRAI Palliative Care assessment was introduced for those in aged residential care diagnosed with less than six months to live . The interRAI Palliative Care assessment is accepted by the Ministry of Health for entry into a residential facility. |
| Switzerland  [81] | The Resident Assessment Instrument – Minimal Dataset (RAI-MDS), is populated from data collected annually (full assessment) and biannually (abbreviated assessment) in nursing homes [76]. While the RAI-MDS has been used in nursing homes since 2000, there are no examples of its application in monitoring the quality of end of life care. |

Supplementary Table 5 Outcome measures collected in the databases/data sources

| Country (database/data source) | Outcome measure collected |
| --- | --- |
| Australia  (Palliative Care Outcomes Collaboration - PCOC) | Palliative Care Phase; Palliative Care Problem Severity Score [95]; Symptom Assessment Scale [96]; Australia-modified Karnofsky Performance Status scale [97]; and Resource Utilisation Groups – Activities of Daily Living [98]. These measurements are taken at the start of a patient's episode of care and at a phase change. |
| Austria | There has been a recent focus on embedding patient-reported outcome measures in palliative care units initially, particularly the Integrated Palliative Care Outcome Scale [99]. Patient-reported outcome measures for pain and shortness of breath have been added to the National Outcomes Measurement project, those are currently not reported upon owing to the lack of data [100]. |
| Denmark  (Danish Palliaitve Care Database - DPD) | European Organisation for Research and Treatment of Cancer Quality of Life Questionaire-Core-15-Palliative Care (EORTC QLQ-C15-PAL) [101] questionnaire, assessing health-related quality of life. Responses to the 15 items and estimated scores for 10 scales: physical function, emotional function, pain, fatigue, nausea/vomiting, dyspnea, lack of appetite, constipation, sleeping difficulties, overall quality of life, are collected. Currently just collected in the day of first contact with SPC or up to 3 days before. It is expected that the next iteration will facilitate collection of follow up measures. |
| Germany  (Nationales Hospiz- und Palliativregister - HOPE) | HOPE-SP-CL (symptom and problem checklist) [102]; Eastern Cooperative Oncology Group (performance status). Additional modules: Minimal Documentation System (self-assessment of the symptoms and the condition of the patient), the German version of the Integrated Palliative Outcome Scale [99] and the basic psycho oncology documentation. |
| Sweden  (Swedish Register of Palliative Care - SRPC) | Data collection is based on the British Geriatrics Society’s statement on what constitutes a good death as an important inspiration and guide [19]. Data includes prevalence and severity of symptoms, prescriptions of essential parenteral drugs as needed, the use of palliative consultation services and bereavement support, whether the death was expected or not, and information provided to patient and family. Once off collection, within one week of the patient's death. |
| Switzerland  (SwissPALL) | SwissPAL, which is in the final stages of development will collect data on entry and exit from SPC services on patient function and symptoms [21-24]. |
| United Kingdom (England)^1^  (The Outcomes and Complexitiy Collaboration (OACC) and the RESOLVE programme) | OACC consists of a suite of six outcome measures for palliative care, three of which are core: Palliative Phase of Illness; Australian-modified Karnofsky Performance Status [97]; Integrated Palliative care Outcome Scale [99].  The project recommends that services implement the suite of measures rather than just a subset, as the combination of all of them is generally much more useful than the use of only one or a couple of the measures alone. The Views on Care is an add-on module to the Integrated Palliative care Outcome Scale [103]. The other two measures are the Zarit Burden Interview and the Barthel Index. It is recommended that the measures are collected on admission to the palliative care service and that there is at least one subsequent assessment, though it's preferable to use ongoing |

Supplementary Table 6 How the databases/data sources are populated

| Country (database/ data source) | Description | Responsible organisation/entity | Frequency |
| --- | --- | --- | --- |
| Australia  (Palliative Care Outcomes Collaboration)  [11] | Clinicians record the clinical assessment information in two ways: (1) using paper forms that are stored with the patient's medical record. Data entry personnel then enter this information into the required ‘phase level’ format, and (2) via direct entry into an electronic patient record. The IT systems then implement an algorithm to convert this assessment level data into ‘phase level’ data. | The national Palliative Care Outcomes Collaboration  office, located within the Australian Health Services Research Institute, University of Wollongong, is responsible for database maintenance and routine reporting and analysis.  Participating specialist palliative care service providers are responsible for populating the database for their respective service. | Twice yearly |
| Austria  (Hospice Austria)  [40, 100, 104] | Services return annual survey data to the hospice and palliative care coordinating organizations via Excel™, or directly into the database. The plausibility of this data is then verified, collated, and returned to Hospice Austria. | Each specialist palliative care service provider collects the survey data. The data are collated and held by Hospice Austria, an independent, non-profit association of around 390 hospice and palliative care institutions in Austria. | Yearly |
| Belgium  (Quality Indicators for Palliaitve Care - QPAC)  [76] | There are four questionnaires, currently completed on paper. For the patients in care, measurements are taken by the patient himself and the care provider involved. For patients who have already died, it is measured with a surviving relative and with the general practitioner (if deceased at home) or with the most involved care provider in the hospital (if deceased in hospital).  Scores of the quality indicators are then automatically calculated for each team, based on a partly automated data processing system in Excel™. | Data collection for the QPAC database is managed by individual service providers.  The QPAC database is under the governance of the VUB-UGent research group 'End-of-Life Care', in collaboration with the Federation Palliative Care Flanders. | Every six months |
| Canada | Canada does not have a national dataset for palliative care. Instead, it makes use of linked administrative datasets. | | |
| Denmark  (Danish Palliative Database - DPD)  [13,14] | Most of the variables in DPD are entered by the specialist palliative care service providers in a web-based data entry system. Two paper-based forms are used for this: a data form consisting of 18 items and a patient-completed questionnaire on palliative care.  The information for completion of the data form is extracted from the medical record, including documents relating to referral, and then transferred to the web-based system. | Data collection for the DPD is the responsibility of individual service providers.  The Clinical Registries in Denmark (of which the DPD is one) are founded on a national initiative, mandated by law and regulated by national government, but financed and owned by regional governments. | Typically after the patient’s death or after the contact has been stopped. |
| Finland | Finland does not have a national palliative care database. It makes significant use of its population and health registries, to evaluate palliative care, predominantly in a research capacity. It also has a well-established benchmarking project to assess the quality of long-term care (Resident Assessment Instrument), which is updated twice yearly, and results in a minimum dataset known as the Terveyden ja hyvinvoinnin laitos (THL RAI) database. | | |
| Germany  (Nationales Hospiz- und Palliativregister - HOPE)  [89, 105] | The patient self-assessment form is completed at every time of documentation when the patient is mentally and physically capable. If the patient cannot fill in the form, the information collected through a patient interview can be obtained from the practitioner or relatives. Typically, data are extracted from the standardised documentation systems of the participating services. The prerequisite for participation is electronic documentation and transmission via a corresponding system that contains the core dataset and has the interface to the Register. | Participating specialist palliative care service providers are responsible for populating the database for their respective service.  Management of the Nationales Hospiz- und Palliativregister is the responsibility of the German Society for Palliative Medicine (DGP). The GDP is a scientific society comprising members from medicine, nursing, and other professional groups. | At least twice in the course of care- at admission and end of care. |
| France | France does not have a national dataset for palliative care. A recently established National Centre for Palliative Care (Centre national des soins palliatifs et de la fin de vie) collects, analyses and publishes data on end of life support and palliative care at the national and regional level. Additionally, researchers make substantive use of the French national health system database (Système National des Données de Santé, SNDS) to provide information on access to, and quality of palliative and end of life care in France. | | |
| Ireland  (Minimum Data Set – MDS)  [41] | Data are submitted by individual specialist palliative care service providers to their Community Healthcare Organisation, via an Excel™ spreadsheet. The CHO submits the validated data to the Business Information Unit Community Healthcare Team for collation and reporting against national and regional key performance indicator targets. | Data collection for the Minimum Dataset is a requirement and the responsibility of individual service providers.  The data are collated and held by the Health Service Executive, the operational arm of Ireland’s publicly funded health system. | Monthly submission |
| Japan  [106-108] | Japan does not have a national palliative care dataset; rather it makes extensive use of existing administrative datasets, and cross-sectional surveys conducted by the Ministry of Health, Labour and Welfare, and other statutory organisations. The Cancer Registry has been in operation in Japan since 2016, and publishes cancer statistics annually. | | |
| New Zealand  (Hospice New Zealand)  [22,109] | Service data, financial data, and community services support data (education and clinical advice and support) are currently collected by an annual data request to all hospice services using a standardised MS Excel™ template. | Hospice New Zealand, an independent national organisation representing all hospice services in New Zealand, coordinates and manages the service data cycle.  Individual service providers share data on these domains with Hospice New Zealand on a voluntary basis. | Data are collected in October for the previous 12-month period to the end of June. |
| Norway  [31] | Despite policy documents recommending the establishment of a national palliative care registry as far back as 2004, Norway does not have a quality register for palliative care on the patient level. The Cancer Registry of Norway has several clinical registries; several of which have started or are starting to include quality of life measures and patient reported outcome measures, e.g. the Breast Cancer Registry and the Colorectal Cancer Registry . | | |
| Sweden  (Svenska palliativregistret - SRPC)  [21, 110-111] | Registration in the SRPC is web-based, but some units first complete a paper version of the ELQ which is later used to complete the web form. All questions have to be answered in the web form before submission. The responsible physician and/or registered nurse at the health care setting of the patient’s death complete the 29-item questionnaire after the patient’s death, using data from medical records and personal knowledge of the patient during the patient’s last week of life. A related party questionnaire is filled in (anonymously), by a close relative after the death has occurred via a secure password protected site on the register\s website. | Data collection is managed at the service-level, with the responsible physician and/or nurse completing forms for each decendent. Additionally, a close relative is invited to complete an anonymous questionnaire.  The SRPC is a national quality registry, financed and operated by local authorities. | Retrospectively, after the patient's death. |
| Switzerland  [22-24] | While Switzerland does not currently have a national dataset for palliative care, a consolidated dataset (SwissPALL) is under review, and a data host for future collaboration offering secured data will shortly be selected. SwissPALL is the efforts of the Swiss Society for Palliative Care, the Swiss Academy of Medical Science and the Federal office of Public Health. | | |
| The Netherlands  [57-59, 91-94] | The recently developed Palliative Care Information System is a government-funded initiative which makes significant use of existing linked data from national datasets to provide a comprehensive description of certain domains of the quality of palliative care. The merged dataset files are stored and accessed through the protected safe environment of Statistics Netherlands. | | |
| United Kingdom England, Northern Ireland, Wales (National Audit of Care at the End of Life - NACEL)  [77-78] | Data for the NACEL is collected during a specified time-period via a bespoke online data entry tool for both the Organisational Level Audit and the Case Note Review.  Excel™ versions of all data specifications are made available for download to assist audit participants with internal data collation prior to the upload of data onto the data collection tool. | All National Health Service acute, community hospital and mental health inpatient providers in England, Northern Ireland and Wales were eligible to register for participation in NACEL. These providers were responsible for completing data collection and returning to NACEL.  The NACEL is managed by the National Health Service Benchmarking Network. | Annual audit |

Supplementary Table 7 Description of governance and validation the databases

| **Country (database/ data source)** | **Governance** | **Validation** |
| --- | --- | --- |
| Australia  (The Palliative Care Outcomes Collaboration - PCOC)  [12, 83, 112] | The national PCOC office, located within the Australian Health Services Research Institute (AHSRI), University of Wollongong, is responsible for the database and routine reporting and analysis. AHSRI is the custodian of the database and owner of the intellectual property. | In order to achieve national consistency, PCOC developed a **data-collection framework** to ensure that the data were useful in understanding quality at a patient, service and health system level.  During data submission, data extracts are loaded into the database for data quality checking. Services receive an error report, and are given the opportunity to amend their data if required. Once corrected the data are again extracted and re-submitted to undergo the same process. This process of error checking may happen multiple times. After the closing date, PCOC undertakes a further data cleaning process before performing analysis and benchmarking and generating individualised reports for participating services. The reports produced every 6 months provide details of data completion. |
| Austria  (Hospice Austria Database)  [100, 104] | Data parameters have been defined by a group of experts and practitioners in hospice and palliative care within the board of Hospice Austria and are re-evaluated regularly. | A first quality check of the data of each of the nine federal states is done by the coordinating organizations. They either check when feeding the data collected via Excel™ sheets in the database or they check what the services themselves have inserted. The database itself comes with certain consistency checks (e.g. checksums).  The second quality check is done by Hospice Austria. All data are checked individually and by comparing the data to the data of the previous year. The results are presented to the coordinating organizations (on a federal state level) and discussed in the board meeting of Hospice Austria.  The final step is a meeting of a representative of the federal state (health/social department) and a representative of the respective federal hospice and palliative care coordinating organization, in which they discuss and agree on the current data set for the federal state. |
| Belgium (QPAC)  [113 – 115] | QPAC is under the governance of the Vrije Universiteit Brussel -UGent research group 'End-of-Life Care', in collaboration with the Federation Palliative Care Flanders. | Validated questions to gather the data for the quality indicators are included in QPAC (where possible). The number of missing responses per question is used as an evaluation criterion to assess the quality indicators. |
| Denmark  (Danish Palliative Care Database - DPD)  [13 – 14; 116] | The Danish Multidisciplinary Cancer Group for Palliative Care forms part of the Danish Clinical Quality Program - National Clinical Registries. The program focuses on preserving the conduct of the Danish databases; improving the use of existing data; standardization of in-, and outputs as well as product- and methodological development. The DPD Secretariat continuously keeps close contact with those who enter the database, and follows up on questions of doubt and other problems via personal contact, manuals and course days. | The DPD secretariat carries out validation and analyses of the data. It carries out logical checks; checks for inconsistencies between date of death and start of treatments; checks for missing registrations when merging with the Danish National Patient Register and follows up on these at the individual units.  Data from the database are continuously validated against the Danish National Patient Register to ensure that all relevant patients are entered in DPD, i.e., whether all patients registered in the Danish National Patient Register as having a contact with an SPC unit are registered in DPD. Data about date of death is validated by linking with the Danish Civil Registration System. The variables in DPD have a high level of data completeness, with completeness at 100% for several variables, reflecting that the majority of the variables are made mandatory when entering into the system. In addition, electronic validations have to be submitted and the form cannot be submitted until all data has been entered. |
| Germany  (Nationales Hospiz- und Palliativregister - HOPE)  [105, 117] | The German Society for Palliative Medicine, together with the German Hospice and Palliative Care Association, has set up a steering group that manages, advises, and develops the Register[113, 118]. |  |
| Sweden  (Swedish Register of Palliative Care - SPRC)  [21, 118] | There is an executive committee and a steering committee with a depth of expertise. These committees visit units to give feedback and information. Monitoring of units is carried out to ensure that registration is conducted and reported in accordance with the register's plan, instructions and applicable laws and regulations. | Sweden's Municipality and Regions have a requirement that all registers must perform validation and monitoring. There are several ways to do so: using logical controls, against source data and against external registers. The SRPC currently performs all three methods. At registration, patient data are initially validated with respect to the accuracy of the personal identity number and the dates entered by the user. Stored data are matched weekly with the central population register and annually with the Cause of Death Register. |
| The Netherlands  Palliative Care Information System (Informatiesysteem Palliatieve Zorg)  [57] | During the development of the Information System, a draft governance structure with two central bodies was drawn up via documentary review, interviews and feedback rounds with national stakeholders from policy, patient advocacy, healthcare practice and research in palliative care. The governance has two central bodies (1) a broadly composed steering group with authority over the Information System, for example over which data are included and in which form it is reported and (2) an advisory committee with experts. Tasks are clearly defined | NA^1^ |
| United Kingdom  (The Outcomes and Complexity Collaboration (OACC) and the RESOLVE programme)  [103] | The OACC project was led by the [Cicely Saunders Institute](https://www.kcl.ac.uk/nursing/departments/cicelysaunders/about/index.aspx) (King’s College London), and is now continued in the RESOLVE programmet led by the [Wolfson Palliative Care Research Centre](https://www.hyms.ac.uk/research/research-centres-and-groups/wolfson-palliative-care-research-centre) (Hull and York Medical School), in collaboration with Hospice UK. | NA^2^ |
| United Kingdom - England, Northern Ireland, Wales  (National Audit of Care at the End of Life - NACEL)  [119] | A multi-disciplinary Steering Group, with input from a wider Advisory Group. A very clear organogram of the NACEL Project Management and Government structure for the audit can be found in the 2020 reports. | Lack of information |

^1^Secondary use of administrative data. ^2^ RESOLVE is working to establish a Palliative Care Outcomes Registry in the UK.

Supplementary Table 8 Evaluating progress towards the implementation of palliative care policy, and to inform development of palliative care services

| Country (database/data source) | Description |
| --- | --- |
| Australia  (The Palliative Care Outcomes Collaboration - PCOC) | National, state and territory, and service-level patient and profile reports are produced twice a year, and made available for review. PCOC is identified as an important source of data in some recent national policy documents: Western Australia End-of-Life and Palliative Care Strategy 2018–2028 [120] and Palliative Care Australia Palliative Care Service Development Guidelines 2018 [121]. |
| Austria (Hospice Austria) | The database of Hospice Austria is used to measure the existing hospice and palliative care service provision against the suggested quality of provision as given in the concept of graded hospice and palliative care in Austria. The graded concept lists how many services of which kind should exist in relation to the population, how the services should be staffed (FTE, professional groups), it lists training prerequisites of the staff and general equipment requirements (e.g. for palliative care units). The monitoring reports describe the quality of hospice and palliative care service provision using data on staff and general number of services of all types and their catchment area [40]. |
| Canada (administrative data) | The Canadian Institute for Health Information (CIHI) published a very detailed description of access to palliative care in 2018, using exiting administrative datasets [47]. The report highlighted the significant differences across the provinces and territories, and the limitations of the data infrastructure to report upon these measures.  The Canadian Partnership against Cancer is leading efforts to collect more pan-Canadian data on palliative care and end-of-life care with the aim of developing a suite of palliative and end-of-life care measures that will lead to better understanding of the palliative and end-of-life experiences of Canadians with cancer and to identify opportunities for system improvement [122, 123]. |
| Denmark  (Danish Palliative Care Database - DPD) | In Denmark, results of quality indicators from DPD are compared against standards set by the DPD Board (in conjunction with clinicians at national and regional level), and compared over time, in an annual report. The results from the 2019 annual report show very large differences regionally and between institutions, and thus provide clear indications of where there is a need for an opportunity for quality improvements within quality indicators [14]. |
| France  (administrative data) | Administrative data was used in an evaluation of the National Plan for the Development of Palliative Care and End-of-Life Support 2015-2018. The evaluation found, that found that despite significant progress being made in palliative care in France, access to end-of-life systems, was implemented in a non-uniform manner across the territories [124].  CNSPFV established in 2016, using a multiple of data sources to produce a '*National Atlas of Palliative Care and End of Life in France*', the most recent being in 2020. It presents demographic and health data which are analysed as finely as possible to highlight the specific features of the department in terms of health care offer but also of patients' needs in their end-of-life trajectories. This mapping is based on renewed indicators  and is part of a time frame which makes it possible to visualize the development and transformation of the health care offer and the needs of patients in relation to this offer [48, 49]. The Atlas reports on Indicators originally derived for the 2018 report as well as additional indicators including number of palliative care beds per 100,000 inhabitants; and distribution of deaths by underlying cause of death in 2016, by sex. |
| Sweden (SPRC) | Sweden's quality registers are used for general planning and management, providing unique opportunities to monitor and improve health care [21]. The main use of the SRPC in policy is to access the quality of care using the indicators and targets set by the National Board of Health and Welfare, and the measurable criteria defined by the SRPC. A yearly report provides detailed description of compliance with indicators and measures and a commentary on same. It highlights the difference in quality of care across different forms of care. For example, the latest report shows that of registered expected deaths in the country, only 30% had symptom's assessed in life final stage in 2019 [118]. |
| The Netherlands  (Informatiesysteem Palliatieve Zorg) | It is envisioned that going forward, the Information System will provide policy-relevant national information on the basis of which the national palliative care policy can be further developed [57]. |
| United Kingdom  (Minimum Data Set;  The Outcomes and Complexity Collaboration (OACC) and the RESOLVE programme) | In the UK, prior to its discontinuation, data from the MDS was used to inform service management; service monitoring and audit; development of local palliative and end of life care strategy and service planning; commissioning of services and development of national policy [23]. For example, it was used to provide trends such as non-cancer activity, death rates in inpatient beds, and demographic patterns of who accesses different services. More recently, data from OACC is used at the population level for commissioning of services, a use that is likely to expand in the future [23]. |
| UK (administrative data)^1^ | England: Public Health England has started publishing *'National profiles of palliative care'*, and an *'Atlas of variation for palliative and end-of-life care in England'*, using administrative data. The profiles developed by the National End-of-life care Intelligence Network, are intended to help local government and health services to improve care at the end of life [63]. The aim of the 'Atlas of variation' series is to highlight geographical variation and to try to differentiate between warranted and unwarranted variation. The Atlas includes maps, charts, time series data and associated statistics presented at different geographical levels for 17 indicators, e.g., place of death, cause of death, hospital activity and number of patients in need of palliative care. These are presented in 29 maps over three sections: the need for palliative and end-of-life care; the role of hospitals in palliative and end-of-life care; and palliative and end-of-life care in the community [65].  Scotland: There has been a recent focus on harnessing existing individual-level administrative datasets to provide the intelligence required for planning palliative care services at various levels (e.g., NHS Board; Health and Social Care Partnerships; Integration Authorities). One measure reported against is 'Location of Death' with information on the number of deaths per year in Scotland and by Integration Authorities, by the following locations: hospital, home, care home and hospice/palliative care units. Further breakdown is available by demographics, long term conditions and causes of death [65].  Wales: A variety of administrative datasets have been used to assess progress made towards actions in a previous strategy, including: average number of admissions, in the 12 months prior to death by cause of death; percentage of SPC referrals with an Advanced Care Plan; percentage of deaths receiving SPC input by age group [67]. |

Currently, there is not an established system to use existing administrative data in Northern Ireland

Supplementary Table 9 How countries use their databases /data sources to apply quality indicators and provide feedback to services

| Countries  (database/data source) | Application of quality indicators | Provision of feedback to individual services |
| --- | --- | --- |
| Australia  (The Palliative Care Outcomes Collaboration - PCOC) | In Australia, the goal for PCOC is to work with services to minimize variation in practice, so as to optimise patient outcomes [125]. The programme aims to improve clinical outcomes in palliative care through an explicit audit and feedback quality cycle. Quality indicators (benchmarks) for the following domains are applied:   - Time from referral to contact - Time in unstable phase - Change in symptoms/problems - Change in symptoms relative to the national average | Each individual PCOC service has [password-protected access to their own service reports](https://apps.ahsri.uow.edu.au/POP/Account/LogOn?ReturnUrl=%2fPOP%2f). Quality Improvement Facilitators work with the service to interpret their data and to initiate clinical change processes where opportunities to improve outcomes through focusing on quality of care are identified [112]. Benchmarking is at a national, jurisdictional or peer group level. Each service may then choose for a period of time to set its targets for improvement in one or more areas of care [23]. |
| Belgium  (QPAC) | There are 31 indicators that evaluate processes and outcomes of care within the following eight domains: physical aspect of care; psychosocial aspects of care; information, communication and care planning with the patient; information, communication and care planning with family; information, communication and care planning between care providers; type of care and circumstances | A report is generated for each facility, with the teams scores from the previous 3 periods and the scores of similar teams (by service type) also reported. [76]. Risk factors that are adjusted for vary by questionnaire but include: age, gender, diagnosis (cancer vs. non-cancer), cognitive function, patient shortage of breath, relationship of the informal caregiver to the patient, length of service, type of care service [76]. |
| Belgium (administrative data) | Population-level indicators for appropriateness of end-of-life care in cancer, chronic obstructive pulmonary disease or Alzheimer’s disease have been derived [45].The indicator sets measure aspects of aggressiveness of care, pain and symptom treatment, SPC, place of care and place of death and coordination and continuity of care. | NA. Population level indicators |
| Canada (administrative data) | Health Quality Ontario and Ontario Palliative Care Network published quality standards for palliative care comprising of 13 quality statements; each of which is accompanied by quality indicators (process, structural, outcomes) [122]. For example, Quality statement 2 is 'People with identified palliative care needs have access to palliative care support 24 hours a day, 7 days a week'. The associated quality indicators are structural (local availability of palliative care support that is accessible 24/7), and outcomes (percentage of people with identified palliative care needs (or their caregivers) who state that they are able to receive community palliative care support, including after-hours care, when needed). | NA. Population level indicators |
| Denmark  (Danish Palliative Care Database - DPD) | In Denmark, the data collected in the DPD supports the estimation of five quality of care indicators:   - % referred, relevant patients who were actually received - % who waited maximally 10 days before admission - % who died from cancer and who obtained contact with a specialist palliative care provider - % screened with a quality of life questionnaire at admission - % discussed at a multidisciplinary conference [13, 14] | The DPD Secretariat, in collaboration with the DPD Board, produces an annual report showing the results of the indicators overall, at the regional level, and at the specialist palliative care unit level. [13, 14] |
| France  (administrative data) | No palliative care quality indicators currently applied | No |
| Finland  (administrative data) | No palliative care quality indicators currently applied | No |
| Germany  (Nationales Hospiz- und Palliativregister - HOPE) | The 'S3-Guideline Palliative care for patients with incurable cancer', sets out 10 quality indicators most of which must be assessed with the screening instruments IPOS or MIDOS, and they signal the use of the National Hospice and Palliative Care Register (DGP Nationales Hospiz- und Palliativregister, for this purpose [126]. | Data are compiled and evaluated according to facility groups. Each facility can access their patient data or the evaluation of their facility in comparison to similar facilities [105].Benchmarking is carried out for the individual facilities, e.g. number and demographic data of patients, diagnoses, symptom situation, drug and non-drug procedures [127]. |
| Ireland | Monthly key performance indicators report against: access to specialist inpatient bed within seven days; percentage of patients triaged within one working day of referral (inpatient unit); access to SPC services in the community provided within seven days, percentage of patients triaged within one working day of referral community); and number of patients who received SPC treatment in their normal place of residence in the month [128]. | There is a lack of information on how data are fed back to the services, or how the data are used by services to improve patient care. |
| Japan  (administrative data) | Work has been undertaken to develop a methodology for measuring the quality indicators of end-of-life care for cancer patients using the Japanese National Database, which was comprised of the health insurance claims data of all Japanese people) [52] | NA. Population level indicators |
| Japan  (surveys) | The Japan Hospice and Palliative care Evaluation (J-HOPE) surveys [72, 73] evaluate the 'quality of care' and 'quality of life' at Palliative Care Units by utilising the Care Evaluation Scale and Good Death Inventory, respectively. | Responses to the surveys are used to provide data to ensure and improve the quality of care provided by participating institutions via feedback based on the results from each institution. |
| Sweden  (Swedish Register of Palliative Care - SRPC) | The data collected in the SRPC supports the estimation of national quality of care indicators, agreed by the National Board of Health and Welfare in 2013, and updated in 2016 [118]. The National Board of Health and Welfare has established national target levels for the indicators (during the last week of life):   - Oral health assessment - Pain assessment - Prescription of opioid for breakthrough pain - Prescribing of as needed anti-anxiety drugs - Without the presence of pressure ulcers - Breakpoint conversation   There are also two other indicators without national target levels – the coverage of the SRPC and the use of symptom assessment tools (other than pain). | Feedback takes place in real time via an interactive output portal [118].  It is possible to use the SRPC output portal to produce results of these indicators, which can be used to provide details of variation in compliance across setting, geographical region and time. |
| Netherlands  (Informatiesysteem Palliatieve Zorg) | There are currently 11 indicators across four domains, that can be reported against [57]:   - Place of death - Use of hospitals care - Use of GP care - Medication prescribed by a doctor   It is expected, with more data, that standards for quality of care, and targets for same, can be developed and implemented. | NA. Population level indicators |
| United Kingdom  The Outcomes and Complexity Collaboration (OACC) and the RESOLVE programme) | The OACC team are involved in the development of case-mix classification, to enable realistic benchmarking of similar services [26]. Benchmarks have recently been established in the RESOLVE project - which are working towards i) an Outcomes Registry for palliative care [22] and ii) benchmarking [129]. | OACC is currently used at the point of care to inform clinical decision making about individual patients; the assessment at the start of spell of care identifies the complexity of needs. OACC is also valuable at the service level to provide aggregated information about residents; to shape and plan services; and for quality assurance. |
| United Kingdom - England, Northern Ireland, Wales  (National Audit of Care at the End of Life) | Nationally agreed quality standards for hospital care at end of life across several themes:   - Recognising the possibility of imminent death - Communication with the dying person - Communication with families and others - Involvement in decision making - Needs of families and others - Individual plan of care/Place of death - Families’ and others’ experience of care - Governance | Each of the themes has a substantial number of indicators associated with it. Within each theme, a summary score is developed and calculated for each hospital to enable easy comparison between hospitals on the different themes. Bespoke dashboards are made available containing a selection of key metrics. All data are anonymised in the online benchmarking toolkit and participating organisations know their own position only. [77-78, 119] |

Supplementary Table 10 Current and future considerations to improve the palliative care infrastructure

| Considerations | Description |
| --- | --- |
| Enhancing the use (and linkage) of existing databases/data sources including clinical data systems | In Australia, until now, it has not been feasible to link the Palliative Care Outcomes Collaboration database with other healthcare data. This issue is being resolved in the next iteration of data collection, where it has been agreed in principal, that there will be identifiers which will facilitate probabilistic linkage to the rest of the health care system [23]. Furthermore, efforts are under way to implement the use of a 'unique identifier' for everyone in the state of New South Wales, which would render it much more feasible to link across the health system, with appropriate ethical approval [23]. The Palliative Care Outcomes Collaboration database has a placeholder for the use of such a unique health identifier.  In Canada, Health Canada's 2018 framework for palliative care [143], and its associated action plan [144], highlighted the collection of data on palliative care as one of four priority areas for action. The plan set out goals of the use of existing big data sources for more than one purpose, and the linkage of existing databases to improve efficiencies in data collection and analysis.  In Northern Ireland the Palliative Care in Partnership Programme is working within the Health and Social Care system on the regional roll-out of a new integrated digital healthcare record (ENCOMPASS) for patients. This new integrated digital information initiative will provide a more comprehensive palliative care information dataset within the hospital and community services and for those patients receiving care in the independent hospice sector [23]. Similarly, work has commenced on developing access to data in primary care under the General Practice Information Platform initiative. This should support improved identification of patients with palliative care needs in the community through enhanced access to information on primary care clinical systems [23].  See further examples from the Netherlands, and Wales in the country narrative summaries (Supplementary File 2). |
| Establishing a new palliative care database/ data source | In New Zealand, Hospice New Zealand is in the process of developing a 'Hospice Data Commons' that will deliver insight and value for hospices across New Zealand. All hospices will collect and contribute common data based on agreed criteria on a regular basis. The information will be stored in a secure common database, and will be regularly analysed using automated systems and information presented back to hospices in a dashboard format. Standards indicators – that relate to the Hospice NZ Standards for Palliative Care 2019, will be applied to the data. Hospice NZ will establish ongoing dedicated data governance and oversight for the common data infrastructure. The aim is to have the first full data upload by hospices commencing the end of 2021 [23].  In Finland, the Ministry of Social Affairs and Health have named a project group to plan a national palliative care database, which is in very early the stages of planning [23].  Finally, several countries are in various stages of developing databases consisting of outcomes measures. See the country level summaries of Switzerland, UK and Wales (Supplementary File 2) for more details. |
| Expanding data capture to include palliative care services in other settings | In Austria, Hospice Austria aims to incorporate basic palliative care in primary care services such as acute hospitals, long-term care facilities, home nursing/home care and general practitioners, and will start to provide data on these endeavours.  In Belgium, QPAC has recently been expanded to and adapted for nursing homes, and these quality indicators will be added to the regional database for palliative care by the Flemish Institute for Quality of Care in 2021.  In Finland, participation in interRAI in nursing home settings will be mandatory from 2023. |
| Enhancing data capture at the service level | *With respect to how services collect and report data*  In Belgium, there is a recommendation that an e-application should be developed (ideally linked to patient' electronic records) to enable services enter data themselves and to immediately request the results of their QPAC [76].  In Sweden, there are plans to improve data capture by transferring data directly from the medical records into the Swedish Register of Palliative Care, which would improve the validity of the data entered in the register, especially as pertains to symptom prevalence.  *With respect to recording changes over time in the patient's condition*  In Denmark, the findings of a research project, where the changes over time in patients' symptoms and problems were analyzed will lead to the development of new indicators based on changes in patients' symptoms and problems over time. Work has been undertaken to provide evidence to assess what variables and indicators will need to be added to enable assessment of changes in patients symptoms and problems over time [23]. |
| Capturing and reporting PROMS and PREMS | In Austria, there is a focus on establishing the systems in palliative care wards to capture and report upon patient reported outcome measures. It is expected that considerations will be given to extending the measures to palliative care teams and inpatient hospices [101].  In Canada, Health Canada's framework and actions plan for palliative care in 2018 [143, 144], set out the goal to develop and promote the use of standardised person- and family-reported outcomes and experience measures, as well as screening and assessment tools across all settings. Furthermore, the Canadian Partnership Against Cancer is leading efforts to collect more pan-Canadian data on palliative care and end-of-life care, and has funded a three-year project to develop a set of indicators for patient reported outcome measures and patient reported experience measures during cancer treatment, including palliative care.  Finally in the United Kingdom, the Outcomes and Complexity Collaboration (and more recently the RESOLVE programme), is working to establish a Palliative Care Outcomes Registry. |
| Developing indicators for palliative care | In Austria, it is expected that when patient reported outcome measures are sufficiently established, the indicators that can be collected based on these will be reported upon (pain and shortness of breath) [40].  In Finland, the Ministry of Social Affairs and Health has commissioned a project on the status of palliative care and convalescent care and the development of quality indicators for palliative care. |
| Improving the methodology of benchmarking of services | In Belgium, effort continue to refine the method used to risk adjustment, to correctly compare teams, and ensure the quality results are transparent to the sector [76]  In the United Kingdom, the Outcomes and Complexity Collaboration (and more recently the RESOLVE programme) are closely involved in the development of case-mix classification, so that, in the longer term, case-mix adjustment of outcome measurement becomes possible, and this will enable realistic benchmarking of similar services useful to services and commissioners alike [26]. Benchmarks have recently been established in the RESOLVE project for quality improvement and bench-marking [130]. |
